# Hitherto-Unnoticed Self-Transmissible Plasmids Widely Distributed among Different Environments in Japan

**DOI:** 10.1128/aem.01114-22

**Published:** 2022-09-07

**Authors:** Masaya Hayakawa, Maho Tokuda, Kensei Kaneko, Koichiro Nakamichi, Yukie Yamamoto, Tatsuya Kamijo, Honoka Umeki, Reimi Chiba, Ryo Yamada, Mitsuya Mori, Kosuke Yanagiya, Ryota Moriuchi, Masahiro Yuki, Hideo Dohra, Hiroyuki Futamata, Moriya Ohkuma, Kazuhide Kimbara, Masaki Shintani

**Affiliations:** a Department of Engineering, Graduate School of Integrated Science and Technology, Shizuoka University, Shizuoka, Japan; b Department of Environment and Energy Systems, Graduate School of Science and Technology, Shizuoka University, Shizuoka, Japan; c Instrumental Research Support Office, Research Institute of Green Science and Technology, Shizuoka University, Shizuoka, Japan; d Japan Collection of Microorganisms, RIKEN BioResource Research Center, Tsukuba, Ibaraki, Japan; e Department of Science, Graduate School of Integrated Science and Technology, Shizuoka University, Shizuoka, Japan; f Green Energy Research Division, Research Institute of Green Science and Technology, Shizuoka University, Shizuoka, Japan; Kyoto University

**Keywords:** plasmid, IncP/P-1, PromA, conjugative transfer, antibiotic resistance gene

## Abstract

Various conjugative plasmids were obtained by exogenous plasmid capture, biparental mating, and/or triparental mating methods from different environmental samples in Japan. Based on phylogenetic analyses of their whole-nucleotide sequences, new IncP/P-1 plasmids that could be classified into novel subgroups were obtained. Mini-replicons of the plasmids were constructed, and each of them was incompatible with at least one of the IncP/P-1 plasmids, although they showed diverse iteron sequences in their *oriV* regions. There were two large clades of IncP/P-1 plasmids, clade I and II. Plasmids in clade I and II included antibiotic resistance genes. Notably, nucleotide compositions of newly found plasmids exhibited different tendencies compared with those of the previously well-studied IncP/P-1 plasmids. Indeed, the host range of plasmids of clade II was different from that of clade I. Although few PromA plasmids have been reported, the number of plasmids belonging to PromAβ, and -γ subgroups detected in this study was close to that of IncP/P-1 plasmids. The host ranges of PromAγ and PromAδ plasmids were broad and transferred to different and distinct classes of *Proteobacteria*. Interestingly, PromA plasmids and many IncP/P-1 plasmids do not carry any accessory genes. These findings indicate the presence of “hitherto-unnoticed” conjugative plasmids, including IncP/P-1 or PromA derivative ones in nature. These plasmids would have important roles in the exchange of various genes, including antibiotic resistance genes, among different bacteria in nature.

**IMPORTANCE** Plasmids are known to spread among different bacteria. However, which plasmids spread among environmental samples and in which environments they are present is still poorly understood. This study showed that unidentified conjugative plasmids were present in various environments. Different novel IncP/P-1 plasmids were found, whose host ranges were different from those of known plasmids, showing wide diversity of IncP/P-1 plasmids. PromA plasmids, exhibiting a broad host range, were diversified into several subgroups and widely distributed in varied environments. These findings are important for understanding how bacteria naturally exchange their genes, including antibiotic resistance genes, a growing threat to human health worldwide.

## INTRODUCTION

**FIG 1:**
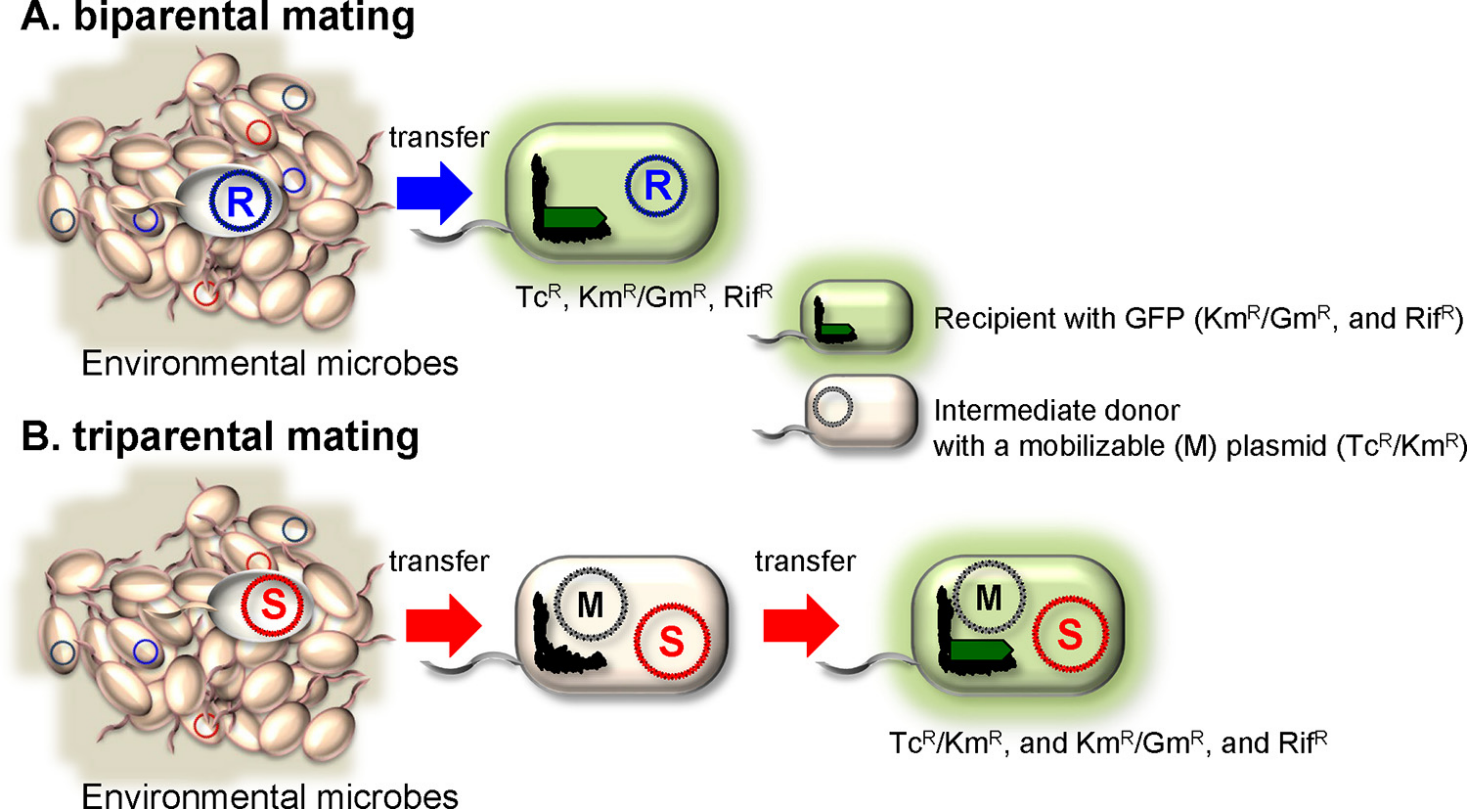
Illustration of the plasmid capture methods used in this study. (A) Biparental mating. Self-transmissible plasmids with antibiotic resistance (Tc was used in this study) genes (R, shown in blue) in environmental microbes are transferred to the prepared GFP-tagged recipient strain (Km or Gm, and Rif resistance). The recipients with R plasmids could be selected on the LB plate with Tc, Km/Gm, and Rif. (B) Triparental mating. Self-transmissible plasmids (S, shown in red) in environmental microbes are transferred to the intermediate donor strain with a mobilizable plasmid (M, shown in black) with an antibiotic resistance gene (for Km or Tc). Then, the S plasmids could be transferred to the GFP-tagged recipient mobilizing the M plasmid. The recipients with S and M plasmids could be selected on the LB plate with Tc or Km and Km or Gm, and Rif. Environmental plasmids are not necessarily always present in the recipient strain in the triparental mating method, and sometimes environmental mobilizable plasmids could be obtained.

Plasmids are extrachromosomal DNA elements, some of which carry a variety of accessory genes, including antibiotic resistance, heavy metal resistance, pathogenic, and catabolic genes, which are transmitted among different bacteria. Because they can promote rapid bacterial evolution and adaptation, as well as the occurrence and spread of multidrug-resistant bacteria, identification of plasmids that are transferred among different bacteria in the natural environment is important. Although there are more than 36,355 plasmids in the National Center for Biotechnology Information (NCBI) database, whose whole nucleotide sequences are available in April 2022 (https://ftp.ncbi.nlm.nih.gov/genomes/GENOME_REPORTS/plasmids.txt), these data do not clearly show which plasmids are transferred in nature. The objective of our study was to identify conjugative plasmids in different environments, including wastewater treatment plants (WWTP), cow manure, lake sediments, marine sediments, paddy sediments, pond sediments, river sediments, and soil. Exogenous plasmid capture methods have been applied to obtain a variety of conjugative plasmids from different microbial communities in environmental samples, including activated sludge, manure, and the rhizosphere (1). Two capture methods have been established: (i) biparental mating, in which a cultivable recipient strain is used to collect conjugative plasmids with specific accessory gene(s), including antibiotic resistance, heavy metal resistance, or metabolic genes; and (ii) triparental mating, in which an intermediate donor with a mobilizable plasmid is used (Fig. 1). The latter method is efficient for collecting conjugative plasmids from the environment because it does not require any marker gene(s) in the plasmid (1). This method depends on the ability of a self-transmissible plasmid in environmental samples to mobilize the prepared mobilizable plasmid (2, 3). The advantage of these methods is that transferable plasmids can be directly obtained independent of the cultivability of their original host bacteria. However, a disadvantage is that the original host bacteria cannot be identified in nature. In triparental methods, the type of plasmid obtained depends on the mobilizable plasmid used (1). New broad-host-range conjugative plasmids have been obtained from various sources, including anaerobic WWTPs, cow manure, and freshwater, using triparental mating (4, 5) with derivatives of pBBR1MCS (6, 7) as mobilizable plasmids. Notably, triparental mating methods can collect conjugative plasmids without accessory genes (4, 5, 8). IncP/P-1 plasmids have been frequently obtained using both methods (5, 8). Triparental mating has yielded plasmids without any accessory genes, and some of these plasmids have been identified as belonging to the incompatibility (Inc) group PromA (4, 5), recently recognized as broad-host-range plasmids (9). These facts indicate exogenous plasmid capture by triparental mating with pBBR1MCS vectors could aid in collecting undetected conjugative plasmids from environmental samples. In this study, we expanded the plasmid sources using various types of environmental samples randomly collected from Japan, and exogenous plasmid capture was performed using both biparental and triparental mating methods.

## RESULTS AND DISCUSSION

### Diverse self-transmissible plasmids were obtained using exogenous plasmid capture.

A total of 1,090 transconjugants were obtained by exogenous plasmid capture (Fig. 1) from 106 different environmental samples, including activated sludge from aerobic WWTPs, anaerobic WWTPs, cow manure, lake sediment, marine sediment, paddy sediment, pond sediment, river sediment, and soil (Table 1; Table S1). Of these, 963 were Pseudomonas resinovorans and 127 were Escherichia coli (Table 1). PCR analyses with specific primer sets for IncP/P-1, PromA, and other previously known plasmids (IncA or C [= IncP-3], IncL or M, IncN, or IncW) revealed that 381 transconjugants possessed these plasmid groups (IncP/P-1, 168; PromA, 213), while at least 618 transconjugants did not exhibit any PCR products with the above primer sets (Table 1). When verifying the presence of plasmids in 553 of the latter “PCR negative” transconjugants, we found that 121 possessed putative plasmids based on plasmid extractions and agarose gel electrophoresis. Nucleotide sequences of the plasmids in “PCR-positive” and “PCR-negative” transconjugants were determined using next-generation sequencing (NGS). A total of 90 complete sequences, including 69 unique plasmid sequences, were obtained (Table S2). Sequence lengths ranged from 2,292 to 149,764 bp. Plasmids smaller than 30 kb were predicted to be mobilizable (pMNBL076-1, pMNBL076-2, pMNBL076-3, pMNCG080-2, pMNCG082-2, and pMNCF093-3) (Table S2). Based on their annotation, a *trfA* gene encoding a replication initiation protein specific to IncP/P-1 plasmids was found in each of the 27 plasmids (see below), whereas the *repA* gene of the PromA plasmids was found in 23 plasmids (two sets of which had identical sequences: pYKCT011-2 and pYKBS026, and pMNCE067 and pMNCK068) (Table S2). The remaining 14 plasmids were identified as plasmids of the IncFII, IncN, IncC/P-3, IncP-9, IncX, and pSN1216-29 groups (Table S2).

**TABLE 1:** Captured plasmids in the present study

Transconjugant	Mating	No. of isolate^*b*^	PCR signals^*a*^							
			IncP/P-1	PromA	pSN1216-29	IncA or C/P-3	IncL and IncM	IncN	IncW	PCR-negative^*c*^
*P. resinovorans*	Triparental	961	142	193	2	0	0	0	0	564
	Biparental	2	2	1	0	0	0	0	0	0
E. coli	Triparental	87	6	18	0	0	0	1	0	45
	Biparental	40	18	1	0	2	0	11	0	9
Total		1,090	168	213	2	2	0	12	0	618

a Some of the PCR signals were found in the same isolates.

b Not all isolates were checked by PCR.

c IncFII, IncP-9, and IncX plasmids were included in the “PCR-negative” plasmids.

### New subgroups of IncP/P-1 plasmids were obtained.

**FIG 2:**
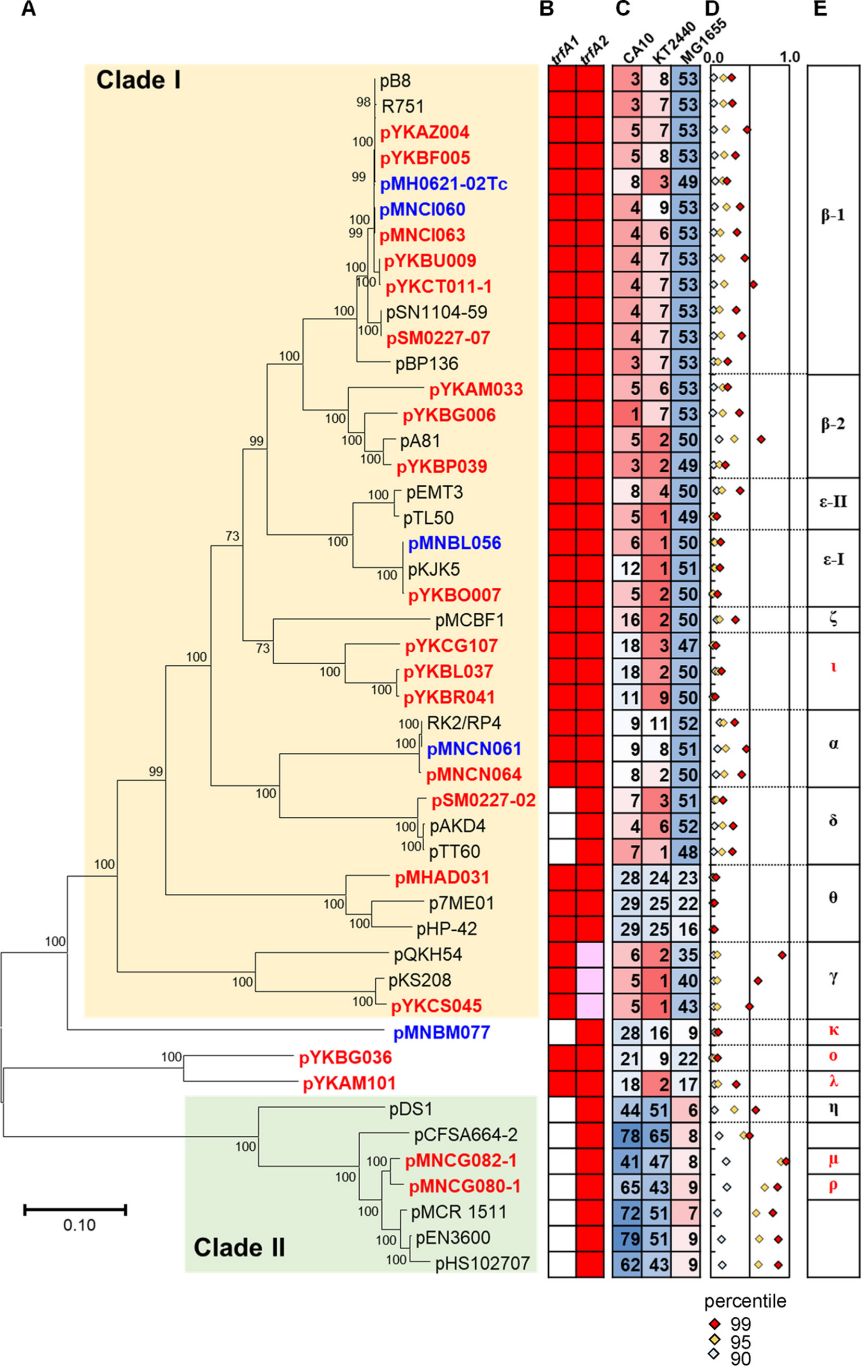
Features of the IncP/P-1 plasmids obtained in this study. (A) Phylogenetic trees of IncP/P-1 plasmids with the concatenated nucleotide sequences of 28 conserved genes (see Table 2), constructed using the maximum likelihood method, with bootstrap percentages at nodes (Tamura-Nei model); plasmids obtained by biparental mating are shown in blue, those obtained by triparental mating are shown in red, and the other reference plasmids are shown in black. The GenBank accession numbers of the reference plasmids are: pB8 (AJ863570), R751 (U67194), pSN1104-59 (AP018709), pBP136 (AB237782), pA81 (AJ515144), pEMT3 (JX469827), pTL50 (MH392238), pKJK5 (AM261282), pMCBF1(AY950444), RK2 (BN000925), pAKD4 (GQ983559), pTT60 (MH392246), p7ME01 (CP006601), pHP-42 (CP001979), pQKH54 (AM157767), pKS208 (JQ432564), pDS1 (KC170283), pCFSA664-2 (CP033354), pMCR_1511 (KX377410), pEN3600 (CP035638), and pHS102707 (KF701335). The solid bar (0.10) shows substitutions per nucleotide position. (B) The presence (red boxes) or absence (white boxes) of *trfA1* and/or *trfA2* are shown. Full-length *trfA2* was not found in IncP/P-1γ plasmids (pink). (C) The rank orders of the distance between each plasmid and chromosomal DNAs of *P. resinovorans* CA10dm4, P. putida KT2440, and E. coli MG1655 are shown by digits and heatmap; higher ranks are shown in red and lower ranks are shown in blue among the 117 reference bacteria (Table S6). (D) The 90, 95, and 99 percentiles of *P* values of each plasmid are plotted (between 0 to 1, high *P* values of close to 1 indicate small Mahalanobis distances and similar 3-mer compositions between a plasmid and chromosome) (Table S6). Names of subgroups are shown, with the newly proposed ones, namely, IncP/P-1ι, κ, ο, μ, λ, and ρ, shown in red.

PCR analyses using previously known primer sets (Table S3) (10) for IncP/P-1 plasmids did not yield positive results for nine plasmids: pMHAD031, pYKBG036, pYKBL037, pYKBR041, pMNBM077, pMNCG080-1, pMNCG082-1, pYKAM101, and pYKCG107. However, they were found to contain coding sequences (CDSs) with a TrfA-conserved domain (NCBI Conserved Domain Database [https://www.ncbi.nlm.nih.gov/Structure/cdd/cdd.shtml] accession number pfam07042). Similarly, each plasmid contained a gene encoding TraI, a relaxase protein involved in conjugative transfer (pfam03432). Phylogenetic analyses of *trfA* and/or *traI* genes and their products showed that some of the obtained plasmids could be assigned to one of the previously known IncP/P-1 subgroups, which have been known as α(alpha), β(beta, β-1, and β-2), γ(gamma), δ(delta), ε(epsilon, ε-I, and ε-II), ζ(zeta), η(eta), and θ (theta) (4, 10–15) (Fig. S1). The other groups were not classified into previously identified subgroups. TrfA exists in two forms, TrfA44 (long form) and TrfA33 (short form) (11). The former is expressed from *trfA1* and the latter is expressed from *trfA2* with separate in-frame translation start sites (11). Notably, only *trfA2* was found in pMNBM077, pMNCG080-1, and pMNCG082-1, similar to IncP/P-1δ, IncP/P-1η, and plasmids in the unidentified subgroup clade (Fig. S1), namely, pCFSA664-2 (16), pMCR_1511 (17), pEN3600 (18), and pHS102707 (19). In contrast, the secondary internal translational start site in the *trfA* gene, encoding the putative short-form TrfA, was not found in pYKCS045 (IncP/P-1γ), as previously reported in other IncP/P-1γ plasmids (20) (Fig. 2). A comparison of the obtained plasmids with other closely related plasmids in the IncP/P-1 group revealed 28 conserved genes, including *trfA*, *trbA*, *trbB*, *trbC*, *trbD*, *trbE*, *trbF*, *trbG*, *trbH*, *trbI*, *trbJ*, *trbL*, *trbN*, *traC*, *traD*, *traE*, *traF*, *traG*, *traI*, *traJ*, *traK*, *traL*, *traM*, *korB*, *korA*, *incC*, *korC*, and *klcA*, with the exception of pMNCI062, which did not possess *traC* or *traD* (Table 2). The phylogenetic trees of concatenated sequences of the 28 genes are shown in Fig. 2 and Fig. S2. Based on these results, novel subgroups of IncP/P-1 plasmids, ι(iota), κ(kappa), ο(omicron), λ(lambda), and μ(mu), were proposed in addition to the previously known subgroups of IncP/P-1 plasmids (Fig. 2). Notably, the concatenated core genes (and *traI* gene) of pMNCG080-1 and pMNCG082-1 were phylogenetically close (Fig. 2; Fig. S2), whereas their *trfA2* and TrfA2 sequences were in different subclades (Fig. S1A and C). These two plasmids had very similar core genes, except for *trfA2* (97% identity at the nucleotide sequence level, excluding *trfA2*), which exhibited a lower identity (64% identity for nucleotide sequences) (Fig. S3). pMNCG080-1 was classified into another subgroup, IncP/P-1ρ(rho) (Fig. 2).

**TABLE 2:** Conserved genes in the IncP/P-1 plasmids^*a*^

	β-1													β-2				ε-II		ε-I			ξ	ι			θ			α			δ			κ	γ			ο	λ	η		µ	ρ			
Gene name	pB8	R751	pYKAZ004	pYKBF005	pMH0621-02Tc	pMNCI060	pMNCI062^*b*^	pMNCI063	pYKBU009	pYKCT011-1	pSN1104-59	pSM0227-07	pBP136	pYKAM033	pYKBG006	pA81	pYKBP039	pEMT3	pTL50	pYKBO007	pKJK5	pMNBL056	pMCBF1	pYKCG107	pYKBL037	pYKBR041	pMHAD031	p7ME01	pHP-42	RK2/RP4	pMNCN061	pMNCN064	pSM0227-02	pAKD4	pTT60	pMNBM077	pQKH54	pKS208	pYKCS045	pYKBG036	pYKAM101	pDS1	pCFSA664-2	pMNCG082-1	pMNCG080-1	pHS102707	pMCR-1511	pEN3600
*trfA*	+	+	+	+	+	+	+	+	+	+	+	+	+	+	+	+	+	+	+	+	+	+	+	+	+	+	+	+	+	+	+	+	+	+	+	+	+	+	+	+	+	+	+	+	+	+	+	+
*ssb*	+	+	+	+	+	+	+	+	+	+	+	+	+	+	+	+	+	+	+	+	+	+	+	+	+	+	+	+	+	+	+	+	−	+	+	−	+	+	+	−	−	+	+	+	+	+	+	+
*trbA*	+	+	+	+	+	+	+	+	+	+	+	+	+	+	+	+	+	+	+	+	+	+	+	+	+	+	+	+	+	+	+	+	+	+	+	+	+	+	+	+	+	+	+	+	+	+	+	+
*trbB*	+	+	+	+	+	+	+	+	+	+	+	+	+	+	+	+	+	+	+	+	+	+	+	+	+	+	+	+	+	+	+	+	+	+	+	+	+	+	+	+	+	+	+	+	+	+	+	+
*trbC*	+	+	+	+	+	+	+	+	+	+	+	+	+	+	+	+	+	+	+	+	+	+	+	+	+	+	+	+	+	+	+	+	+	+	+	+	+	+	+	+	+	+	+	+	+	+	+	+
*trbD*	+	+	+	+	+	+	+	+	+	+	+	+	+	+	+	+	+	+	+	+	+	+	+	+	+	+	+	+	+	+	+	+	+	+	+	+	+	+	+	+	+	+	+	+	+	+	+	+
*trbE*	+	+	+	+	+	+	+	+	+	+	+	+	+	+	+	+	+	+	+	+	+	+	+	+	+	+	+	+	+	+	+	+	+	+	+	+	+	+	+	+	+	+	+	+	+	+	+	+
*trbF*	+	+	+	+	+	+	+	+	+	+	+	+	+	+	+	+	+	+	+	+	+	+	+	+	+	+	+	+	+	+	+	+	+	+	+	+	+	+	+	+	+	+	+	+	+	+	+	+
*trbG*	+	+	+	+	+	+	+	+	+	+	+	+	+	+	+	+	+	+	+	+	+	+	+	+	+	+	+	+	+	+	+	+	+	+	+	+	+	+	+	+	+	+	+	+	+	+	+	+
*trbH*	+	+	+	+	+	+	+	+	+	+	+	+	+	+	+	+	+	+	+	+	+	+	+	+	+	+	+	+	+	+	+	+	+	+	+	+	+	+	+	+	+	+	+	+	+	+	+	+
*trbI*	+	+	+	+	+	+	+	+	+	+	+	+	+	+	+	+	+	+	+	+	+	+	+	+	+	+	+	+	+	+	+	+	+	+	+	+	+	+	+	+	+	+	+	+	+	+	+	+
*trbJ*	+	+	+	+	+	+	+	+	+	+	+	+	+	+	+	+	+	+	+	+	+	+	+	+	+	+	+	+	+	+	+	+	+	+	+	+	+	+	+	+	+	+	+	+	+	+	+	+
*trbK*	+	+	+	+	+	+	+	+	+	+	+	+	+	+	+	+	+	+	+	+	+	+	+	+	+	+	+	+	+	+	+	+	+	+	+	+	+	+	+	+	+	−	+	+	+	+	−	+
*trbL*	+	+	+	+	+	+	+	+	+	+	+	+	+	+	+	+	+	+	+	+	+	+	+	+	+	+	+	+	+	+	+	+	+	+	+	+	+	+	+	+	+	+	+	+	+	+	+	+
*trbM*	+	+	+	+	+	+	+	+	+	+	+	+	+	+	+	+	+	+	+	+	+	+	+	+	+	+	+	+	+	+	+	+	+	+	+	−	+	+	+	+	+	+	+	+	+	+	+	+
*trbN*	+	+	+	+	+	+	+	+	+	+	+	+	+	+	+	+	+	+	+	+	+	+	+	+	+	+	+	+	+	+	+	+	+	+	+	+	+	+	+	+	+	+	+	+	+	+	+	+
*trbP*	+	+	+	+	+	+	+	+	+	+	+	+	+	+	+	+	+	+	+	+	+	+	+	+	+	+	+	+	+	+	+	+	+	+	+	+	+	+	+	+	+	+	−	+	+	+	+	+
*upf30.5*	+	+	+	+	+	+	+	+	+	+	+	+	+	+	+	+	+	−	+	−	+	−	+	+	−	−	+	+	−	−	−	−	+	−	+	−	−	−	+	−	−	−	−	−	−	−	−	−
*traC*	+	+	+	+	+	+	−	+	+	+	+	+	+	+	+	+	+	+	+	+	+	+	+	+	+	+	+	+	+	+	+	+	+	+	+	+	+	+	+	+	+	+	+	+	+	+	+	+
*traD*	+	+	+	+	+	+	−	+	+	+	+	+	+	+	+	+	+	+	+	+	+	+	+	+	+	+	+	+	+	+	+	+	+	+	+	+	+	+	+	+	+	+	+	+	+	+	+	+
*traE*	+	+	+	+	+	+	+	+	+	+	+	+	+	+	+	+	+	+	+	+	+	+	+	+	+	+	+	+	+	+	+	+	+	+	+	+	+	+	+	+	+	+	+	+	+	+	+	+
*traF*	+	+	+	+	+	+	+	+	+	+	+	+	+	+	+	+	+	+	+	+	+	+	+	+	+	+	+	+	+	+	+	+	+	+	+	+	+	+	+	+	+	+	+	+	+	+	+	+
*traG*	+	+	+	+	+	+	+	+	+	+	+	+	+	+	+	+	+	+	+	+	+	+	+	+	+	+	+	+	+	+	+	+	+	+	+	+	+	+	+	+	+	+	+	+	+	+	+	+
*traH*	+	+	+	+	+	+	+	+	+	+	+	+	+	+	+	+	−	−	−	−	+	−	+	+	−	−	−	−	−	+	+	+	−	+	−	−	+	+	−	−	−	−	−	−	−	−	−	−
*traI*	+	+	+	+	+	+	+	+	+	+	+	+	+	+	+	+	+	+	+	+	+	+	+	+	+	+	+	+	+	+	+	+	+	+	+	+	+	+	+	+	+	+	+	+	+	+	+	+
*traJ*	+	+	+	+	+	+	+	+	+	+	+	+	+	+	+	+	+	+	+	+	+	+	+	+	+	+	+	+	+	+	+	+	+	+	+	+	+	+	+	+	+	+	+	+	+	+	+	+
*traK*	+	+	+	+	+	+	+	+	+	+	+	+	+	+	+	+	+	+	+	+	+	+	+	+	+	+	+	+	+	+	+	+	+	+	+	+	+	+	+	+	+	+	+	+	+	+	+	+
*traL*	+	+	+	+	+	+	+	+	+	+	+	+	+	+	+	+	+	+	+	+	+	+	+	+	+	+	+	+	+	+	+	+	+	+	+	+	+	+	+	+	+	+	+	+	+	+	+	+
*traM*	+	+	+	+	+	+	+	+	+	+	+	+	+	+	+	+	+	+	+	+	+	+	+	+	+	+	+	+	+	+	+	+	+	+	+	+	+	+	+	+	+	+	+	+	+	+	+	+
*kfrC*	+	+	+	+	+	+	+	+	+	+	+	+	+	+	+	+	+	+	+	+	+	+	+	+	+	+	+	+	+	+	+	+	+	+	+	+	+	+	+	+	+	+	+	−	+	+	+	+
*kfrB*	+	+	+	+	+	+	+	+	+	+	+	+	+	+	+	+	+	+	+	+	+	+	+	+	+	−	+	+	+	+	+	+	+	+	−	+	+	+	+	+	+	+	+	+	+	+	+	−
*kfrA*	+	+	+	+	+	+	+	+	+	+	+	+	+	+	+	+	+	+	+	+	+	+	+	+	+	+	+	+	+	+	+	+	+	+	+	+	+	+	+	+	+	−	−	+	−	−	−	−
*korB*	+	+	+	+	+	+	+	+	+	+	+	+	+	+	+	+	+	+	+	+	+	+	+	+	+	+	+	+	+	+	+	+	+	+	+	+	+	+	+	+	+	+	+	+	+	+	+	+
*korA*	+	+	+	+	+	+	+	+	+	+	+	+	+	+	+	+	+	+	+	+	+	+	+	+	+	+	+	+	+	+	+	+	+	+	+	+	+	+	+	+	+	+	+	+	+	+	+	+
*incC*	+	+	+	+	+	+	+	+	+	+	+	+	+	+	+	+	+	+	+	+	+	+	+	+	+	+	+	+	+	+	+	+	+	+	+	+	+	+	+	+	+	+	+	+	+	+	+	+
*kleE*	+	+	+	+	+	+	+	+	+	+	+	+	+	+	+	+	+	+	+	+	+	+	+	+	+	+	+	+	+	+	+	+	+	+	+	+	+	+	+	+	+	+	+	+	+	−	+	−
*kleB*	+	+	+	+	+	+	+	+	+	+	+	+	−	−	−	−	−	+	+	+	+	+	+	−	−	−	−	−	−	+	+	+	−	−	−	−	−	−	−	−	−	−	−	+	+	−	+	−
*kleA*	+	+	+	+	+	+	+	+	+	+	+	+	+	+	+	+	+	+	+	+	+	+	+	+	+	+	+	+	+	+	+	+	+	+	+	−	−	−	−	+	+	−	−	+	−	−	−	−
*korC*	+	+	+	+	+	+	+	+	+	+	+	+	+	+	+	+	+	+	+	+	+	+	+	+	+	+	+	+	+	+	+	+	+	+	+	+	+	+	+	+	+	+	+	+	+	+	+	+
*klcB* ^*c*^	+	+	+	+	+	+	+	+	+	+	+	+	+	+	+	+	+	+	+	+	+	+	+	+	+	+	+	+	+	+	+	+	+	+	+	+	+	+	+	+	+	+	+	+	+	+	+	+
*klcA*	+	+	+	+	+	+	+	+	+	+	+	+	+	+	+	+	+	+	+	+	+	+	+	+	+	+	+	+	+	+	+	+	+	+	+	+	+	+	+	+	+	+	+	+	+	+	+	+

a The conserved genes are shaded.

b pMNCI062 did not carry *traCD* genes.

c *klcB* is not conserved in the related plasmids, pS228 or pQKH54.

### Accessory genes, including antibiotic resistance genes, are carried by transposons and/or integrons in IncP/P-1 plasmids.

Several IncP/P-1 plasmids identified (12/27) contained antibiotic resistance genes (Table 3). Although antibiotic resistance gene(s) were not used as a marker to select self-transmissible plasmids in triparental mating, the plasmids obtained by exogenous plasmid capture through biparental mating and triparental mating carried the resistance gene(s) (Table 3; Supplemental Text S1). Putative resistance genes for tetracycline (*tetA*, *tetC*, *tetG*, and *tetX*), beta-lactams, including carbapenems (*bla_AER-1_*, *bla_NPS_*, *bla_OXA-1_*, *bla_OXA-101_*, *bla_IMP-1_*, *bla_GES_*, and *bla_SHV-173_*), aminoglycosides (*aac*, *aad*, *aph*, and *str*), and erythromycin (*ereA*) were identified, and the hosts carrying these plasmids were resistant to the tested antibiotics (Table 3). Hosts with plasmids carrying resistance genes for tetracycline and/or beta-lactams showed higher resistance to the corresponding antibiotics than those without plasmids (Table 3). These genes were carried by other mobile genetic elements, including Tn*402*-like transposons or class 1 integrons (21) (Table 3; Fig. S4), indicating that they could spread among different replicons. Notably, antibiotic resistance genes were only found in activated sludge from WWTPs and/or urban rivers, lakes, or marine sediments, but not in soil samples (Table 3). This was probably because the former samples were contaminated with some antibiotics, including sulfonamides and macrolides, although their concentrations were rather low (<1 mg/L) (22–24).

**TABLE 3:** IncP/P-1 plasmids obtained in this study

Name	Inc_group	Size (bp)	Source	Antibiotic resistance genes and the resistances of the hosts (R, resistance, S, sensitive, ND, not determined)^*a*^										
				Accessory genes	Tc (12.5)^*b*^	Gm (30)	Ap (50)	Km (50)	Km (25)	Km (12.5)	Cm (30)	Sm (50)	Sm (25)	Em (25)
pYKBP039	IncP/P-1β-2	55,596	Activated sludge	Tn*402*-class1 integron (*bla_OXA_*, *bla_GES_*, *aac(6′)-Ib*, *ere(A)*, *bla_IMP_*, *aac(6′)-Ib*)	ND	S	R	S	R	R	ND	ND	ND	R
pYKBO007	IncP/P-1ε-I	42,530	Activated sludge	Transposon	ND	ND	ND	ND	ND	ND	ND	ND	ND	ND
pYKBR041	IncP/P-1ι	45,846	Activated sludge	*aac(6′)-31*	ND	ND	ND	R	R	R	ND	ND	ND	ND
pYKCG107	IncP/P-1ι	46,366	Activated sludge	Transposon	ND	ND	ND	ND	ND	ND	ND	ND	ND	ND
pMNCG080-1	IncP/P-1ρ	69,454	Activated sludge	No accessory	ND	ND	ND	ND	ND	ND	ND	ND	ND	ND
pMNCG082-1	IncP/P-1μ	74,115	Activated sludge	*bcs*	ND	ND	ND	ND	ND	ND	ND	ND	ND	ND
pSM0227-07	IncP/P-1β-1	51,612	Anaerobic WWTP	Tn*402* with *mgtAC*	ND	ND	ND	ND	ND	ND	ND	ND	ND	ND
pSM0227-02	IncP/P-1δ	47,983	Anaerobic WWTP	*dam, alwI*	ND	ND	ND	ND	ND	ND	ND	ND	ND	ND
pMH0621-02Tc	IncP/P-1β-1	64,795	Lake sediment	*sul2*, *tet(X6)*, *tetG mer* operon, Tn*21*-like transposon	R	ND	ND	ND	ND	ND	ND	ND	ND	ND
pMHAD031	IncP/P-1θ	42,885	Lake sediment	No accessory	ND	ND	ND	ND	ND	ND	ND	ND	ND	ND
pMNCN061	IncP/P-1α	64,346	Marine sediment	*tetAR*, *strAB*, *bla_SHV_*, *aph(3′)-Ib*	R	S	R	ND	ND	ND	ND	S	R	ND
pMNCN064	IncP/P-1α	68,925	Marine sediment	*tetAR*, transposon (Tn*402*-class1 integron *[qacH, aadA1, sul1], mer* operon), *aph(3′)-Ib*	R	S	ND	R	R	R	ND	S	S	ND
pYKBF005	IncP/P-1β-1	60,124	River sediment	Tn*402*-class1 integron (*qacE*Δ-*sul1-orf5, aac[6′]-IIa*, *aadA6*, *bla_OXA_*), Tn (*relE*)	ND	S	R	S	S	R	ND	S	S	ND
pYKBU009	IncP/P-1β-1	53,186	River sediment	Tn*501*(*mer* operon), IS*1071*	ND	ND	ND	ND	ND	ND	ND	ND	ND	ND
pYKCT011-1	IncP/P-1β-1	57,620	River sediment	Tn*501(*remnant) (*mer* operon), IS*21*, Tn*3* family (*bla_NPS_*), *relE*	ND	ND	R	ND	ND	ND	ND	ND	ND	ND
pMNCI060	IncP/P-1β-1	56,363	River sediment	Transposon (*aph[3′]-Ia*, *tetCR*, *strAB*), *tetAR*	R	S	R	ND	ND	ND	ND	R	R	ND
pMNCI062	IncP/P-1β-1	38,628	River sediment	No accessory	ND	ND	ND	ND	ND	ND	ND	ND	ND	ND
pMNCI063	IncP/P-1β-1	52,178	River sediment	*aroA*	ND	ND	ND	ND	ND	ND	ND	ND	ND	ND
pYKCS045	IncP/P-1γ	49,370	River sediment	Tn*402*-class1 integron (like) (*bla_GES-5_*, *aac[6′]-Ib*)	ND	S	R	S	S	R	ND	ND	ND	ND
pMNBL056	IncP/P-1ε-I	52,432	River sediment	Class1 integron (*dfrB1*, *qacEΔ*-*sul1*-*orf5*, *tetRA*)	R	S	S	ND	ND	ND	ND	ND	ND	ND
pYKBL037	IncP/P-1ι	64,506	River sediment	Transposon (*strAB*, class1 integron(*aac(6′)-Ib*, *ere(A)*, *qacEΔ*-*sul1*-*orf5*). Transposon (*bla_AER_*, *qacL*)	ND	S	R	R	R	R	ND	S	R	R
pMNBM077	IncP/P-1κ	53,339	River sediment	Transposon (*tetAR*, transposon)	R	S	S	ND	ND	ND	ND	ND	ND	ND
pYKAZ004	IncP/P-1β-1	58,771	Soil	Tn*402*-class1 integron (*qacE*Δ-*sul1-orf5*), IS*1071*	ND	ND	ND	ND	ND	ND	ND	ND	ND	ND
pYKBG006	IncP/P-1β-2	51,488	Soil	IS*1071*	ND	ND	ND	ND	ND	ND	ND	ND	ND	ND
pYKAM033	IncP/P-1β-2	42,722	Soil	No accessory	ND	ND	ND	ND	ND	ND	ND	ND	ND	ND
pYKBG036	IncP/P-1ο	43,728	Soil	*ompA*	ND	ND	ND	ND	ND	ND	ND	ND	ND	ND
pYKAM101	IncP/P-1λ	69,067	Soil	Two ISs	ND	ND	ND	ND	ND	ND	ND	ND	ND	ND

a Tc, tetracycline; Gm, gentamicin; Ap, ampicillin; Km, kanamycin; Cm, chloramphenicol; Sm, streptomycin; Em, erythromycin.

b The digits in parentheses indicate the concentration of each antibiotic (μg/mL).

### IncP/P-1 group plasmids can be classified into two clades.

IncP/P-1 group plasmids could be divided into two groups: clade I and clade II (Fig. 2). The former included subgroups, namely, IncP/P-1α, β, γ, δ, and ε, and the plasmids of these subgroups exhibit a broad host range (10–15, 25). However, the host ranges of plasmids belonging to ζ and θ subgroups and one of the newly proposed subgroups, IncP/P-1ι, have not been clearly identified (10–15). Clade II contained the newly identified plasmids pMNCG080-1 (IncP/P-1μ) and pMNCG082-1 (IncP/P-1ρ), as well as pDS1 (IncP/P-1η) (4), and other plasmids in unidentified subgroups (Fig. 2). Plasmids in the other three new subgroups, IncP/P-1κ, IncP/P-1ο, and IncP/P-1λ, were located between these two clades (Fig. 2).

Incompatibility tests between mini-replicons of several IncP/P-1 plasmids (IncP/P-1β-1, γ, -η, -ι, -κ, -ο, -θ, -μ, -λ, and -ρ) were performed to determine whether these plasmids were incompatible. We found that mini-pBP136 in clade I was incompatible with mini-pDS1 (clade II) and other mini-plasmids in clade I (mini-pYKCG107, mini-pMHAD031, and mini-pYKCS045) (Table 4). Although mini-pMNBM077 was incompatible with mini-pBP136, the other four plasmids, mini-pYKBG036, mini-pYKAM101, mini-pMNCG080-1, and mini-pMNCG082-1, were compatible with mini-pBP136 (Table 4). Interestingly, mini-pYKBG036 was incompatible with mini-pYKCG107, mini-pYKAM101, and mini-pMNCG080-1, whereas mini-pYKAM101 was incompatible with mini-pYKCG107 and mini-pYKBG036 (Table 4). Mini-pMNCG082-1 was incompatible with mini-pMHAD031 and mini-pDS1, whereas mini-pMNCG080-1 was incompatible with mini-pYKBG036 (Table 4). These facts indicate the plasmids obtained in the present study (shown in Fig. 2) were incompatible with at least one of the IncP/P-1 plasmids, and they could all be recognized as members of IncP/P-1 plasmids. Each of them could be classified into different subgroups of IncP/P-1 plasmids, but not all were incompatible with each other. One of the newly found clade II plasmids, pMNCG080-1, showed compatibility with the other clade II plasmids, pMNCG082-1 and pDS1, but was incompatible with pYKBG036 (Table 4). Considering pMNCG080-1 and pMNCG082-1 had highly conserved core genes, except for *trfA2* (Fig. S3), the replicons of pMNCG080-1 might be replaced by different types of IncP/P-1 plasmids, similar to pYKBG036 (IncP/P-1ο) (Fig. S5). Here, clade II was not recognized as a group that included incompatible plasmids, but as a group of plasmids with conserved core genes (Fig. 2). It should be noted our incompatibility tests might have overlooked weak incompatibility because we used mini-plasmids containing only *trfA* operon with its promoter and *oriV*, which did not necessarily show the same copy number as their original plasmids. It was, therefore, possible that incompatible plasmids could be misidentified as compatible. Thus, plasmids showing a slightly higher transformation efficiency than the incompatible plasmids but lower than the compatible plasmids were recognized as incompatible (I* in Table 4). For more in detailed analyses for the incompatibility, copy numbers should be compared between each mini-plasmid and its original plasmid. Additional incompatibility tests using original plasmids will be also important in the future.

**TABLE 4:**
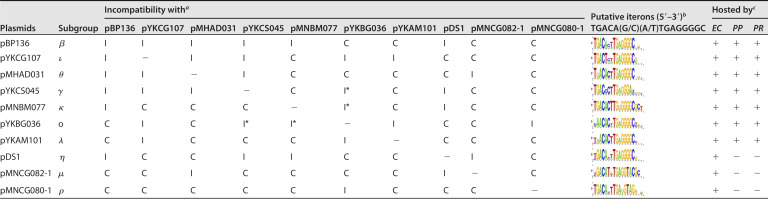
Incompatibility of IncP/P-1 plasmids and their host ranges

a “I” indicates the E. coli DH5α transformants showing resistance to Km and Tc were not detected (below detection limit <8.4 × 10^1^ CFU/μg DNA, which was the efficiency by mini-pBP136_Km and mini-pBP136_Tc). “C” indicates that the transformation efficiency by two “compatible” mini-plasmids are >2.3 × 10^3^ CFU/μg DNA. “I*” indicates that the two mini-plasmids did not show strict incompatibility (the frequency was not lower than that by incompatible plasmids but much lower _[around 102 CFU/μg DNA]_ than that by compatible plasmids). The transformation efficiency of each single mini-replicon was more than 2.1 × 10^5^ CFU/μg-DNA.

b Consensus sequence of iterons were created by WebLogo (57).

c “+” indicates that transformants or transconjugants of the plasmid were obtained for each derivative strains of E. coli MG1655 (“*EC*”), P. putida KT2440 (“*PP*”), and/or *P. resinovorans* CA10dm4R (“*PR*”), while “–” indicates no transformants of the mini-replicons were detected (below detection limits, <0.67 to 1.0 × 10^3^ CFU/μg-DNA).

This phenotype of incompatibility is due to the similarity of TrfA and iterons in *oriV*, which are multiple small DNA repeats that interact with TrfA (26, 27). The alignments of amino acid residues in the partial domains of TrfA-interacting iterons (28) with putative iteron sequences in each subgroup are shown in Fig. S5 and Table S4. The pairwise distances of TrfA proteins from 49 different IncP/P-1 plasmids are shown in Table S5. The distances between compatible plasmids (shown in Table 4) were not necessarily larger than those between incompatible plasmids, especially those between pMHAD31 and pDS1 (compatible, 0.340) or between pYKCG107 and pYKBG36 (incompatible, 0.754) (Table S5). In contrast, the important domains of TrfA protein in RK2/RP4 (28), the DNA binding domain (DBD), and winged helix-turn-helix (WH) motifs 1 and 2 (WH1 and WH2) were substituted with different residue(s) in the four plasmids (pYKBG036, pYKAM101, pMNCG080-1, and pMNCG082-1) compatible with pBP136 (Fig. S5; Table S4). As for the iterons, the second, ninth, and 11th to 14th guanines and the 14th and 15th cytosines in iterons 5′-TGACA(G/C)(A/T)TGAGGGGC-3′ are important for the interactions with TrfA of RP4/RK2 (27), and these nucleotides are well-conserved in those in pBP136 (Table 4). Among the above four plasmids, the second guanine was replaced with adenine in pYKAM101 and pYKBG036 (Table 4). Furthermore, in comparison with pMNCG082-1, the 13th and 14th cytosines were replaced with thymine and adenine, respectively, and the 15th cytosine was replaced with guanine in pMNCG080-1 (Table 4). These facts indicate that the second, 13th, and 14th guanines may be critical for the interaction between TrfA of pBP136 and the four plasmids. However, the conserved nucleotides in the iterons were not necessarily important for the incompatibility of several plasmids; those in some iterations were conserved, but the plasmids were compatible, including pYKCG107, pDS1, pMNCG082-1, and pMNCG080-1, whereas those in others were not conserved, but the plasmids were incompatible, including pYKBG036 and pMNCG080-1 or pDS1 and pMNCG082-2 (Table 4). These facts imply the interaction(s) between TrfA and its iterons may be different from those of RP4/RK2. Further in-depth molecular-level analyses will be necessary to understand their incompatibility.

### IncP/P-1 group plasmids in clade II showed different host ranges than those in clade I.

To assess differences in the host ranges of these plasmids, filter mating assays with different recipients or electroporation with IncP/P-1 mini-plasmids were performed. Plasmids in clade I were transferred from E. coli to P. putida and/or *P. resinovorans* or vice versa (the frequency was 10^−4^–10^−1^ per donor) (Table 4). In contrast, no Pseudomonas transconjugants of plasmids in clade II, including pDS1, were detected (Table 4), which coincided with previous reports that pDS1 cannot be transmitted to bacteria other than E. coli, including Pseudomonas (4). Plasmids pYKBG036(IncP/P-1ο), pMNBM077(IncP/P-1κ), and pYKAM101(IncP/P-1λ) could be transferred from E. coli to P. putida and *P. resinovorans*. Notably, pMNCG080-1 and pMNCG082-1 mobilized pBBR1MCS-3 into Pseudomonas strains at a low frequency (10^−7^ per donor). This indicated their conjugative transfer systems could transfer genetic material from E. coli to strains of the genus Pseudomonas, but the plasmids might not be replicated in these strains. Mini-replicons of pMNCG080-1, pMNCG082-1, and pDS1 were introduced into E. coli and P. putida via electroporation. Neither of the mini-replicons could be replicated in P. putida (<0.67 to 1.0 × 10^3^ CFU/μg-DNA) while both could in E. coli (3.3 to 7.7 × 10^3^ CFU/μg-DNA), suggesting that their replication host ranges could be narrower than their conjugation host ranges. In summary, clade II plasmids of IncP/P-1, including pDS1, pMNCG080-1, and pMNCG082-1, exhibited different host ranges from clade I plasmids (Table 4).

Suzuki et al. reported nucleotide compositions, including GC content and oligonucleotide signatures, exhibit greater similarity to their host chromosomes for narrow-host-range plasmids than for broad-host-range plasmids (29). It has also been reported the Mahalanobis distance (dissimilarity of oligonucleotide compositions) of trinucleotide composition and its *P* value could be used to distinguish between narrow-host-range and broad-host-range plasmids (30). Therefore, the Mahalanobis distances of trinucleotide composition were calculated between each plasmid and the chromosomal DNA of the reference bacteria (117 strains; Table S6). The rank order of Mahalanobis distances between IncP/P-1 plasmids and chromosomal DNAs was found to differ among different subgroups (Table S6). Fig. 2C shows the rank order of the distance between each plasmid and chromosomal DNA of *P. resinovorans* CA10dm4, P. putida KT2440, and E. coli MG1655. The rank orders of these three strains were significantly different between clades I and II. Except for IncP/P-1θ, the plasmids in clade I were ranked higher for CA10dm4 and KT2440 than for MG1655 (Fig. 2C). In contrast, the plasmids in clade II exhibited higher ranks for MG1655 than for the other two strains (Fig. 2C). *P* values close to 1 for distance indicate small Mahalanobis distances and similar 3-mer compositions between a plasmid and chromosome (29, 30). The 90, 95, and 99 percentiles of *P* values for each plasmid are shown in Fig. 2D. Notably, the 95 and 99 percentiles of *P* values for pMNCG082-1 and pMNCG080-1 were higher than the others, whereas those of IncP/P-1ε-I (pMNBL056 and pYKBO007), IncP/P-1ι (pYKCG107, pYKBL037, pYKBR041), IncP/P-1θ (pMHAD031), pMNBM077, and pYKBG036 were lower (Fig. 2D). These facts suggest pMNCG082-1 and pMNCG080-1 may have narrower host ranges than others. Other plasmids, including IncP/P-1θ plasmids and the novel plasmids, IncP/P-1ι, pMNBM077, and pYKBG036 may have broad but different host ranges from the well-studied IncP/P-1 plasmids in clade I, IncP/P-1α, β, δ, and ε (10–15, 25). In conclusion, the host range of IncP/P-1 plasmids in clades I and II may be different, and further experimental comparisons of their host ranges should be performed.

### PromA plasmids without accessory genes were obtained by exogenous plasmid capture.

**FIG 3:**
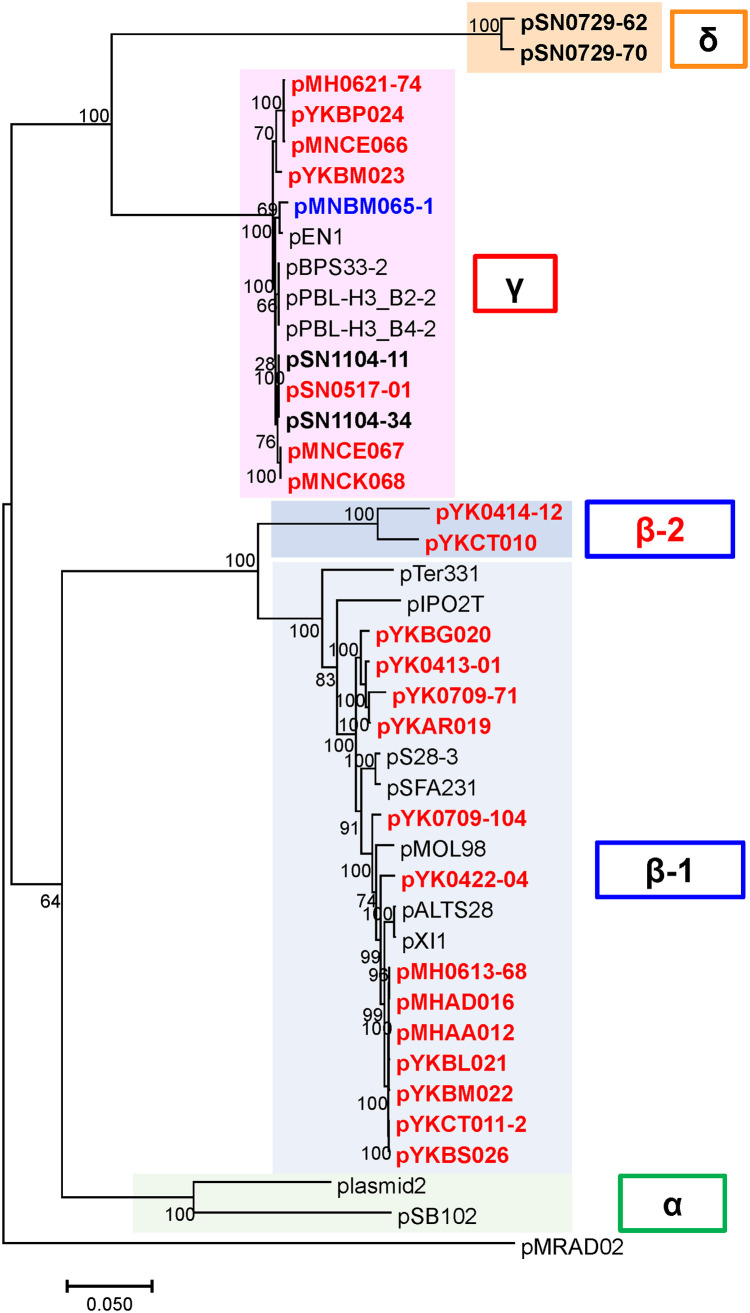
Phylogenetic trees of PromA plasmids. Phylogenetic trees of PromA plasmids with concatenated nucleotide sequences of 24 conserved plasmid genes, constructed using the maximum likelihood method, with bootstrap percentages at nodes (Tamura-Nei model). The plasmid obtained by biparental mating is shown in blue, while those obtained by triparental mating are shown in red, and the other reference plasmids are shown in black (the plasmids obtained in our previous study are shown in bold). The GenBank accession numbers of the reference plasmids are: pSN0729-62 (AP018705), pSN0729-70 (AP018706), pEN1 (MN536506), pBPS33-2 (CP044551), pBPL-H3_B2-2 (CP044977), pBPL-H3_B4-2 (CP044974), pSN1104-11 (AP018707), pSN1104-34 (AP018708), pTer331 (EU315244), pIPO2T (AJ297913), pS28-3 (MF495477), pSFA231 (KJ850907), pMOL98 (FJ666348), pALTS28 (MN366357), pXI1 (CP020047), plasmid2 (CP009154), pSB102 (AJ304453), and pMRAD02 (CP001003). PromAβ-2 is proposed in this study.

Among the plasmids obtained, 23 possessed genes similar to those of the PromA plasmids. A previous study identified 24 conserved genes in the PromA plasmids (5). These genes were also conserved in all PromA plasmids obtained in this study. Phylogenetic analyses of the concatenated 24 genes showed the plasmids could be classified into the PromAβ (15 plasmids) and PromAγ (8 plasmids) subgroups (Fig. 3; Fig. S6). There were two clades of PromAβ (Fig. 3; Fig. S6). One of them included two plasmids, pYK0414-12 and pYKCT010, whereas the others contained the previously known PromAβ plasmids (Fig. 3; Fig. S6). The PromAβ group can be divided into two new sub-subgroups: PromAβ-1 and PromAβ-2 (Fig. 3; Fig. S6). Based on the phylogenetic tree, pMRAD02 may not be a PromAα member (Fig. 3; Fig. S6). No accessory genes were found in any of the PromA group plasmids (Fig. S7). Currently, there are a few plasmids in the PromA group carrying accessory genes: in PromAα, pSB102 with mercury resistance genes (31); in PromAβ-1, pALTS28 with multidrug resistance genes (32) and pS28-3 with a florfenicol resistance gene (33); and in PromAγ, four plasmids, pPBL-H3_BS2-2, pPBL-H3_BS4-2, pBPS33-2, and pEN1 with linuron degradative genes (34). The genetic structures of PromA plasmids with accessory genes were reported to be unstable (34), which could be one of the reasons why the obtained PromA plasmids did not possess any accessory genes.

### PromA mini-plasmids containing *repA* and *oriV* could be replicated in strains of different *Proteobacteria*.

Gstalder et al. identified a mini-replicon DNA region in pMOL98, a PromAβ plasmid (35). They identified the origin of replication as 19-bp repeats (iterons) and a 27-bp AT-rich region adjacent to a 29-bp GC-rich region (35). Based on these reports, mini-plasmids with the putative *repA* and *oriV* regions were constructed as representative plasmids for the pMH0613-68 (PromAβ-1), pYK0414-12 (PromAβ-2), pSN1104-11 (PromAγ), and pSN0729-62 (PromAδ) subgroups. All mini-plasmids were replicated in E. coli and P. putida. Putative genes related to plasmid replication, encoding primase and/or single-strand DNA-binding protein (*ssb*), were found both in PromAγ and PromAδ (but not in PromAβ-1 or PromAβ-2) plasmids upstream and downstream of the putative *oriV* region (Fig. S8). These genes were not required for the replication of mini-replicons, at least in these hosts. The putative iterons to which the RepA protein might bind were conserved in all PromA plasmids, namely, the 17-bp repeats (5′-CGCTGAAacTGTCTTGC-3′, repeated three times in PromAα, four times in PromAβ-1, PromAβ-2, and PromAγ, and five times in PromAδ) (Fig. S8), although the amino acid sequences of RepA were not necessarily highly conserved (54% to 80% identity) (Table S7). Because the PromAγ and PromAδ plasmids were incompatible with each other (data not shown), it is possible the iterons could be similarly recognized by RepA of the PromA group plasmids.

### The PromAγ and PromAδ plasmids showed a broad host range.

Previously, pSN1104-11 (PromAγ) and pSN0729-62 (PromAδ) were transferred to strains of different classes of *Proteobacteria*, indicating these are broad-host-range plasmids (5). In this study, filter mating assays for these two plasmids were performed using bacterial communities extracted from soil and/or cow manure as recipients. Transconjugants belonging to different classes of *Proteobacteria* were obtained (Table S8), indicating both plasmids were broad-host-range plasmids. For both plasmids, many transconjugants belonged to *Gammaproteobacteria* (Table S8). Interestingly, *Alphaproteobacteria* strains were obtained as transconjugants of pSN1104-11, whereas *Betaproteobacteria* strains were obtained as transconjugants of pSN0729-62 (Table S8), suggesting the host ranges of PromAγ and PromAδ may be different. This was expected because the two plasmids exhibited a 10% variation in G+C content (64% for PromAγ plasmids and 54% for PromAδ plasmids) (5). Therefore, as the nucleotide compositions of plasmids, including G+C content, could be affected by those of host bacteria (30, 36), as shown in the case of IncP/P-1 plasmids, it is possible plasmids in these two subgroups are hosted by different kinds of bacteria.

### The plasmids were distributed in different environmental samples.

Exogenous plasmid capture methods are not necessarily appropriate for understanding the abundance and/or distribution of plasmids in nature because the number of plasmids obtained is limited. Bacterial strains of intermediate donors and recipients, selective marker(s) for biparental mating, and mobilizable plasmids for triparental mating could change the results of capture (1). Thus, to assess the abundance and distribution of plasmids, PCR-Southern blot analyses with specific primers and probes for IncP/P-1 (clade I or II) and PromA (α to δ) were performed for total community DNA (TC-DNA) extracted from 113 different environmental samples collected from various sites (47 sites) in Japan. As shown in Table 5, Table S1, and Table S2, IncP/P-1 (subgroups of clade I) was found in 26 different types of samples, including those collected from activated sludge, anaerobic WWTP, lake sediments, marine sediments, pond sediments, river sediments, and soil (Table 5). Smalla et al. identified a clade I group of IncP/P-1 plasmids in different types of environmental samples, showing this plasmid group is widely distributed in nature as vehicles for different genes with a broad host range (37, 38). Notably, PromA group plasmids were identified in 24 different types of environmental samples, including activated sludge, anaerobic WWTP, cow manure, lake sediments, marine sediments, paddy sediments, pond sediments, river sediments, and soil (Table 5). These results indicate the PromA group plasmids and plasmids in clade I of the IncP/P-1 group were widely distributed in nature. Interestingly, plasmids in clade II of the IncP/P-1 group were found at four sites: activated sludge, pond, and river sediments (Tables 5; Table S1). Considering these environmental samples were collected from metropolitan areas, the newly found IncP/P-1 plasmids in clade II may have spread among different aqueous environments that are closely related to human activity. The plasmids in clade II of IncP/P-1 group are “vehicles” of important antibiotic resistance genes among *Enterobacteriaceae*; a carbapenem-resistance gene, *bla*_IMP-8_ and *bla*_KPC-2_, was found in pEN3600 (18) and pHS102707 (19), respectively, whereas a colistin resistance gene, *mcr-1*, was found in pMCR_1511 (17). These plasmids were identified as IncP/P-1 group plasmids in PlasmidFinder 2.1 (https://cge.cbs.dtu.dk/services/PlasmidFinder/) (39), although they did not exhibit high sequence identity with other IncP/P-1 plasmids in clade I and were only found in *Enterobacteriaceae*. One of the features of these plasmids is that they possess only the *trfA2* gene as a replication-initiation protein. Yano et al. showed TrfA1 is more detrimental to the persistence of plasmids than TrfA2 because it activates the host helicase DnaB and increases the copy number of plasmids (40). These functions are important for the promiscuity of IncP/P-1 plasmids containing TrfA1, but not for stability in some bacteria (40). Therefore, it is possible the plasmids in IncP/P-1 clade II might possess only TrfA2 to enhance their stability in specific hosts of *Enterobacteriaceae*.

**TABLE 5:** Distribution of IncP/P-1 and PromA plasmids in different environmental samples^*a*^

Environmental samples	IncP/P-1^*b*^										PromA
	Clade I					- - -			Clade II		-
	αβε	ι	δ	θ	γ	κ	ο	λ	η	ρμ	αβγδ
Activated sludge (4WWTPs, 20 samples)	11(14)	8(2)	1(0)	0(0)	1(0)	0(0)	0(0)	ND(0)	0(0)	1(2)	5(6)
Anaerobic WWTP (2 samples)	ND(1)	ND(0)	ND(1)	ND(0)	ND(0)	ND(0)	ND(0)	ND(0)	ND(0)	ND(0)	ND(1)
Cow manure (1 sample)	0(0)	0(0)	0(0)	0(0)	0(0)	0(0)	0(0)	ND(0)	0(0)	0(0)	1(0)
Lake sediment (17 lakes, 21 samples)	0(3)	0(0)	0(21)	0(1)	0(1)	0(0)	0(0)	ND(0)	0(0)	0(0)	7(67)
Marine sediment (2 sites, 10 samples)	0(2)	0(0)	0(0)	0(0)	0(0)	0(0)	0(0)	ND(0)	0(0)	0(0)	0(0)
Paddy sediment (1 sample)	0(0)	0(0)	0(0)	0(0)	0(0)	0(0)	0(0)	ND(0)	0(0)	0(0)	1(0)
Pond sediment (3 samples)	0(4)	0(0)	0(8)	0(0)	0(0)	0(0)	0(0)	ND(0)	0(0)	1(0)	1(0)
River sediment (9 rivers, 40 samples)	0(7)	0(1)	2(11)	0(0)	0(1)	0(1)	0(0)	ND(0)	0(0)	2(0)	6(39)
Soil (10 sites, 17 samples)	1(19)	0(0)	1(9)	0(0)	1(0)	0(0)	0(1)	ND(1)	0(0)	0(0)	3(2)
Total (47 sites, 113 samples)	12 (50)	8(3)	4(50)	0(1)	2(2)	0(1)	0(1)	0(1)	0(0)	4(2)	24(115)

a The digits indicate the number of environmental samples showing positive signals with the specific probe in PCR-Southern blot analyses, whereas those in parentheses indicate the number of isolates with the plasmid (Tables S1 and S2). ND, not determined.

b Newly proposed subgroups of IncP/P-1 group are shown in bold. -, indicates IncP/P-1 plasmids not included by clades I or II.

### Conclusion.

In the present study, we found “hitherto-unnoticed” self-transmissible plasmids, including new subgroups of IncP/P-1 and PromA. This was likely owing to several plasmids that possessed no previously known accessory genes (or unstable genes) that could endow the hosts with specific phenotypes. The reason the plasmids obtained in the present study did not possess any accessory genes remains unclear. It is possible that accessory genes may have been lost during the exogenous capture procedure because they are a burden for some hosts. Nonetheless, these “vacant vehicles” could still participate in the exchange of various types of genes, including antibiotic resistance genes, among different bacteria in nature.

Exogenous plasmid capture methods are efficient for collecting unnoticed conjugative plasmids from environmental samples. Nevertheless, these methods still have bias owing to the type of recipient strain and/or mobilizable plasmids used. Although several novel plasmids were successfully obtained in the present study, the plasmids were closely related to one another because they could all be replicated in recipient cells for bi- and triparental mating and/or mobilize pBBR1 plasmids in triparental mating. Thus, these methods do not necessarily capture the most frequently transferred or the most abundant plasmids in the microbial community. Different recipients or mobilizable plasmids should be used to collect undetected conjugative plasmids. Another limitation of the exogenous plasmid capture method is that the original host(s) of the obtained plasmids cannot be identified. Notably, only a few PromA plasmids (7/42) were found in their host bacteria, suggesting that the original hosts of most PromA plasmids might not be easily cultivated. Other limitations of the present study include the lack of information on how IncP/P-1 or PromA plasmids are diversified in different environments. To understand the roles of these newly discovered plasmids and their diversification history, their host ranges and transfer routes in different environments should be elucidated. Further in-depth analyses are required to understand the features of these plasmids, including their stability, copy number, and fitness cost, in the absence or presence of accessory genes in different host strains. These insights will shed light on the routes of horizontal gene transfer among different microbes.

## MATERIALS AND METHODS

### Bacterial strains, plasmids, and culture conditions.

The bacterial strains and plasmids used in the present study are listed in Table 6. Escherichia and Pseudomonas strains were cultivated in Luria broth (LB) (41) at 30°C or 37°C. R2A plates containing 1.5% agar were used for filter matings. Ampicillin (Ap, 50 μg/mL), chloramphenicol (Cm, 30 μg/mL), erythromycin (Em, 25 μg/mL), kanamycin (Km, 30 μg/mL for plasmid capture and 50 μg/mL for the other experiments), gentamicin (Gm, 30 μg/mL), rifampicin (Rif, 30 μg/mL for plasmid capture and 50 μg/mL for the others), streptomycin (Sm, 25 or 50 μg/mL), and tetracycline (Tc, 12.5 μg/mL for E. coli and 50 μg/mL for the others) were added to the medium. Cycloheximide (100 μg/mL) was added to prevent fungal growth. For plate cultures, LB was solidified using 1.5% agar (wt/vol).

**TABLE 6:** Bacterial strains and plasmids used in this study

Strains or plasmids	Relevant characteristics	Reference
Bacterial strains		
*Escherichia coli*		
DH5α	F^−^, *endA1*, *hsdR17*(r_k_^−^, m_k_^−^), *phoA*, *supE44*, *thi-1*, λ^−^, *recA1*, *gyrA96*, *relA1*, Δ(*lacZYA- argF*)U169, ψ80d*lacZ*ΔM15	RBCBioscience
JM109	F' [*tra*D36, *proAB*, *lacI*^q^, *lacZ*ΔM15], *recA1*, *endA1*, *gyrA96*, *thi-1*, *hsdR17*(r_K_^−^ m_K_^+^), e14^−^ (*mcrA*^−^), *supE44*, *relA1*, Δ(*lac*-*pro*AB)	RBCBioscience
MG1655	F^−^, λ^−^, *rph-1*	58, 59
MG1655R	Spontaneous Rif^r^ strain of MG1655	This study
MG1655RG	MG1655, Rif^r^, mini-Tn*5*-Gm^r^ gene was inserted in the chromosome	This study
MG1655RK	MG1655, Rif^r^, mini-Tn*10*-Km^r^ gene was inserted in the chromosome	This study
MG1655RT	MG1655, Rif^r^, mini-Tn*10*-Tc^r^ gene was inserted in the chromosome	This study
MG1655RGFP	MG1655, Rif^r^, mini-Tn*5*-Km-P*_A1/O4/O3_*-RBSII-gfpmut3*-T_0_-Cm^r^-T_1_ was inserted in 578584 nt in the chromosome (accession no. NC_000913)	This study
S17-1λ*pir*	Tm^r^, Sm^r^, *recA*, *thi*, *pro*, *hsdR^−^M*^+^, RP4: 2-Tc:Mu: Km Tn*7, λpir*	55
* Pseudomonas putida*		
KT2440	pWW0-free *Pseudomonas putida* mt-2	60
KT2440G	Derivative strain of KT2440, Gm^r^ gene is inserted into PP_4780 (NC_021505, see Supplemental text S1)	This study
SMDBS	A *dapB*-deleted strain of SM1443, Rif^r^ of KT2440 (KT2442) with mini-Tn*5*-*lacI*^q^ cassette inserted into the chromosome	25
SMDBS(pSN1104-11*gfp*Tc)	SMDBS harboring pSN1104-11*gfp*Tc	This study
SMDBS(pSN0729-62::*gfp*)	SMDBS harboring pSN0729-62::*gfp*	This study
*P. resinovorans* CA10dm4R	Derivative strain of CA10dm4 spontaneously Rif^r^.	61
*P. resinovorans* CA10dm4RGFP	CA10dm4R, miniTn*7*(Gm) P*_A1/O4/O3_ gfp-a* was inserted into chromosome, just downstream of *glmS* gene (6265580 nt, NC_021499) (Gm^r^, Cm^r^).	5
Plasmids		
mini-pBP136_Km	Km^r^, DNA region containing *ssb, trfA, oriV* of pBP136	This study
mini-pBP136_Tc	Tc^r^, DNA region containing *ssb, trfA, oriV* of pBP136	This study
mini-pYKCG107_Tc	Tc^r^, DNA region containing *ssb, trfA, oriV* of pYKCG107	This study
mini-pMHAD031_Km	Km^r^, DNA region containing *ssb, trfA, oriV* of pMHAD031	This study
mini-pMHAD031_Tc	Tc^r^, DNA region containing *ssb, trfA, oriV* of pMHAD031	This study
mini-pYKCS045_Km	Km^r^, DNA region containing *ssb, trfA, oriV* of pYKCS045	This study
mini-pYKCS045_Tc	Tc^r^, DNA region containing *ssb, trfA, oriV* of pYKCS045	This study
mini-pMNBM077_Km	Km^r^, DNA region containing *ssb, trfA, oriV* of pMNBM077	This study
mini-pMNBM077_Tc	Tc^r^, DNA region containing *ssb, trfA, oriV* of pMNBM077	This study
mini-pYKBG036_Km	Km^r^, DNA region containing *trfA, oriV* of pYKBG036	This study
mini-pYKBG036_Tc	Tc^r^, DNA region containing *trfA, oriV* of pYKBG036	This study
mini-pYKAM101_Km	Km^r^, DNA region containing *trfA, oriV* of pYKAM101	This study
mini- pYKAM101_Tc	Tc^r^, DNA region containing *trfA, oriV* of pYKAM101	This study
mini-pDS1_Km	Km^r^, DNA region containing *ssb, trfA, oriV* of pDS1	This study
mini-pDS1_Tc	Tc^r^, DNA region containing *ssb, trfA, oriV* of pDS1	This study
mini-pMNCG080-1_Km	Km^r^, DNA region containing *ssb, trfA, oriV* of pMNCG080-1	This study
mini-pMNCG080-1_Tc	Tc^r^, DNA region containing *ssb, trfA, oriV* of pMNCG080-1	This study
mini-pMNCG082-1_Km	Km^r^, DNA region containing *ssb, trfA, oriV* of pMNCG082-1	This study
mini-pMNCG082-1_Tc	Tc^r^, DNA region containing *ssb, trfA, oriV* of pMNCG082-1	This study
mini-pMH0613-68_Tc	Tc^r^, DNA region containing *repA*, *oriV* of pMH0613-68	This study
mini-pSN0729-62_Tc	Tc^r^, DNA region containing *repA*, *oriV* of pSN0729-62	This study
mini-pSN1104-11_Tc	Tc^r^, DNA region containing *repA*, *oriV* of pSN1104-11	This study
mini-pYK0414-12_Tc	Tc^r^, DNA region containing *repA*, *oriV* of pYK0414-12	This study
pBBR1MCS-2	Km^r^, *lacZα mob*; compatible with IncP, IncQ, and IncW plasmids	7
pBBR1MCS-3	Tc^r^, *lacZα mob*; compatible with IncP, IncQ, and IncW plasmids	7
pBBR1MCS-5	Gm^r^, *lacZα mob*; compatible with IncP, IncQ, and IncW plasmids	7
pBSL202	Ap^r^, Gm^r^ mini-Tn*5*	62
pJBA28	Ap^r^, Km^r^, delivery plasmid for mini-Tn*5*-Km-P*_A1/O4/O3_*-RBSII-*gfp*mut3^*^-T_0_- Cm^r^-T_1_	54
pK18_1104-11	pK18mobsacB with homologous DNA region with pSN1104-11 (1-kb DNA regions upstream and downstream of the target site) and Tc^r^ and P*_A1/O4/O3_*-RBSII-gfpmut3* cassette	This study
pK18mobsacB	pMB1, *lacZα*, *mob*, *kan*, *sacB*	63
pRK2013	ColEI replicon, a helper plasmid for RP4/RK2 *oriT*-mediated conjugative transfer; Km^r^	64
pSN0729-62	PromAδ plasmid	5
pSN0729-62::*gfp*	Mini-Tn*5*-Km-P*_A1/O4/O3_*-RBSII-gfpmut3*-T_0_-Cm^r^-T_1_ cassette in 35612 nt of pSN0729-62 (AP018705)	This study
pSN1104-11	PromAγ plasmid	5
pSN1104-11*gfp*Tc	Tc^r^ and P*_A1/O4/O3_*-RBSII-gfpmut3* cassette in 37978 nt of pSN1104-11 (AP018707)	This study

### Collection of environmental samples.

Environmental samples were randomly collected from different sites in Japan. Twenty activated sludge samples were collected from four WWTPs in different cities in Japan. Anaerobic WWTP samples were collected from a lab-scale upflow anaerobic sludge blanket reactor for methane fermentation, as previously described (5). Cow manure was collected from cows that were not fed antibiotics in the Sumiyoshi Field of Miyazaki University, Japan, on June 28 and 30, 2020. Other environmental samples were collected from different locations in Japan, including lake sediments, marine sediments, paddy sediments, pond sediments, river sediments, and soil (57 sites, 117 samples; Table S1).

### Exogenous plasmid capture.

Exogenous isolation of plasmids was performed as previously described (5). Green fluorescent protein (GFP)-tagged recipient E. coli MG1655RGFP or *P. resinovorans* CA10dm4RGFP was used (Fig. 1; Table 6; Text S1). For biparental mating, tetracycline was used as a selection marker for conjugative plasmids (i.e., a conjugative plasmid with tetracycline resistance genes was obtained). In contrast, an intermediate donor strain E. coli with pBBR1MCS-2 or pBBR1MCS-3 (7) was used for triparental mating. One gram (wet weight) of each environmental sample potentially containing helper bacterial cells with self-transmissible plasmids was resuspended in 10 mL phosphate-buffered saline (PBS). The overnight-cultured donor and recipient strains were mixed with the above environmental samples with a helper strain on a 0.22 μm pore size membrane filter (ADVANTEC, Dublin, CA, USA) in LB containing cycloheximide for 48 h at 30°C (filter mating). Afterward, the mixture on the filter was collected and resuspended in 5 mL of PBS, and 100 μL of a serial dilution was spread on LB with Rif, Km, and Gm. Colonies on medium with green fluorescence were isolated and subjected to genetic analyses.

### DNA manipulations.

Total DNA from the bacterial strains was extracted using a NucleoSpin Tissue Kit (TaKaRa Bio, Shiga, Japan). Total DNA from the isolates was extracted and purified using an AcroPrep Advance 96 Filter Plate (Pall Life Sciences, Westborough, MA, USA) as previously described (5) or using NucleoSpin 96 Flash (TaKaRa Bio). Small plasmids were extracted from E. coli using the NucleoSpin Plasmid EasyPure Kit (TaKaRa Bio). Alkaline lysis extraction was performed as previously described (42), followed by agarose gel electrophoresis to confirm the presence of plasmids. Large plasmids were extracted using the Large Construct Kit (Qiagen, Hilden, Germany). PCR was performed on a T100 thermal cycler (Bio-Rad, Hercules, CA, USA) using the KOD One PCR Master Mix (TOYOBO, Osaka, Japan). Primers used in this study are listed in Table S3. The amplification conditions except for PCR-Southern blot analyses were as follows: 30 cycles at 98°C for 10 s, 55°C for 5 s, and 68°C for 5 s/1 kb amplicon. Restriction enzymes (New England Biolabs Inc., Ipswich, MA, and/or TaKaRa Bio), HiYieldTM Gel/PCR DNA fragment extraction kit (RBC Bioscience Corp., New Taipei City, Taiwan), NEBuilder Hifi DNA Assembly System (New England Biolabs), and competent E. coli JM109 and DH5α cells (RBC Bioscience) were used to clone DNA fragments. Plasmids were introduced into different bacterial strains by electroporation using a MicroPulser electroporator (Bio-Rad Laboratories Inc.). All other procedures were performed according to standard methods (41).

### Plasmid sequencing and annotation.

The nucleotide sequences of IncP/P-1 or PromA group plasmid DNA were determined using MiSeq, HiSeq250 platform (Illumina, San Diego, CA), and PacBio RS II System (PacificBiosciences, Menlo Park, CA, USA). The detailed methods are described in the Supplemental Text. The first annotations were performed using DFAST-core v.1.2.5 (43) with an in-house database of plasmid sequences collected from NCBI RefSeq, and then corrected manually. The annotated genes of the IncP/P-1 plasmids were reannotated and named according to those in R751 (12), except for *kfrB* (upf54.8, R751) and *kfrC* (upf54.4, R751). Similarly, those in the PromA plasmid were annotated using pSN1104-11 (5). Accessory genes, including putative metabolic and/or transporter genes, were subjected to BLAST analysis (https://blast.ncbi.nlm.nih.gov/Blast.cgi) to identify similar sequences. Genotypic screening of antibiotic resistance genes in these plasmids was performed using the Comprehensive Antibiotic Resistance Database (CARD) (44).

### Phylogenetic analysis of plasmids obtained.

Plasmid maps were visualized using SnapGene (http://www.snapgene.com/). The core genes of the IncP/P-1 and PromA group plasmids were determined by comparative analyses of the plasmids using Easyfig ver. 2.2.2 (45). The nucleotide or amino acid sequences of genes in plasmids were aligned using ClustalW (46), and the neighbor-joining method (47) (Kimura 2-parameter method [48] for nucleotide sequences and Poisson correction method [49] for amino acid sequences), maximum likelihood method (Tamura-Nei model [47] for nucleotide sequences and JTT matrix-based model [50] for amino acid sequences), and minimum-evolution method (51) (Kimura 2-parameter method [48] for nucleotide sequences and Poisson correction method [49] for amino acid sequences) were used for the unrooted trees using MEGA 7 (52). Other *in silico* analyses were performed using Geneious Prime 2021 (53).

### Measuring distance of trinucleotide composition in a plasmid and chromosome.

Refseq chromosome accessions for reference prokaryotic genomes were retrieved from the NCBI genome list. There were 120 genomes in the “prok_reference_genomes.txt” file, downloaded from https://ftp.ncbi.nlm.nih.gov/genomes/GENOME_REPORTS/ on August 24, 2020. Among these, 116 genomes were available (Table S6). The chromosomes of Pseudomonas resinovorans CA10dm4 (NC_021499), which were not included in the prokaryotic genomes mentioned above, were experimentally used as recipients for exogenous plasmid capture. If multiple chromosomes were present, only the largest primary chromosome was retained in the analysis, as previously described (54). The 3-mer compositions of IncP/P-1 plasmids were compared with those of 117 prokaryotic chromosomal DNA (a set of nonoverlapping 5-kb chromosomal segments) using previously described methods (29, 30, 54). The smaller the Mahalanobis distance, the more similar the 3-mer compositions of the plasmid and chromosome. The Mahalanobis distance was converted to an empirical *P* value ranging from 0 (minimal similarity) to 1 (maximal similarity), as previously described (30). High *P* values close to 1 indicate small Mahalanobis distances and similar 3-mer compositions between a plasmid and a chromosome (29, 30, 54).

### Incompatibility testing.

Transformation of E. coli DH5α competent cells (RBC Bioscience) with two mini-replicons was performed, one of which had a Km resistance gene, and the other had a Tc resistance gene (Table 6). When no transformants or only a few small colonies were detected on LB plates with Km and Tc (below detection limits <8.4 × 10^1^ CFU/μg DNA), the two plasmids were recognized as incompatible. Mini-pBP136_Km and mini-pBP136_Tc (IncP/P-1β) were used as controls for incompatible plasmids and mini-pBP136_Km and mini-pSN1104-11_Tc (PromAγ) were used as controls for compatible plasmids. The former showed <8.4 × 10^1^ CFU/μg-DNA, while the latter showed 1.9 × 10^4^ CFU/μg-DNA.

### Preparation of *gfp*-tagged plasmids.

A mini-Tn*5* with *P_A1/O4/O3_*-RBSII-gfpmut3* and Km-resistance gene on pJBA28 (54) was introduced into plasmid pSN0729-62 (5) using E. coli S17-1λ*pir* (55) as described previously (56). For pSN1104-11*gfp*Tc, *P_A1/O4/O3_*-RBSII-gfpmut3* on pJBA28 were introduced into the plasmid by homologous recombination (Text S1).

### Filter mating assays for PromA group plasmids.

The donor strains P. putida SMDBS (pSN1104-11*gfp*Tc) and P. putida SMDBS (pSN0729-62::*gfp*) were precultured in LB medium with Tc or Km. Microbes in environmental samples, including soil and cow manure, were used as recipient bacteria. The soil sample was collected at Shizuoka University, Hamamatsu, Japan (34.73N 137.72E) on July 5, 2019. The microbial fraction was extracted from 40 g of soil as previously described (25). Cow manure was sampled from cows that were not fed antibiotics in the Sumiyoshi Field of the University of Miyazaki, Japan, on November 7, 2018. The number of microbial cells in the extracted samples was counted by microscopy after staining the cells with 4′,6-diamidino-2-phenylindole (DAPI) or SYBR green. Mating between the donor and recipient bacteria (microbes extracted from soil samples or 1 g of cow manure) was performed as follows: 1 mL of overnight culture of the plasmid donor in LB medium was harvested, washed with PBS, and then suspended in PBS. Approximately 10^8^ CFU/mL of the donor suspended in 130 μL of PBS was mixed with 130 μL of 10^8^ to 10^9^ cells/mL bacteria extracted from the above environmental samples. The sample mixture was dropped on 0.45 μm pore-size filters. Three different filter matings were performed to collect diverse transconjugants: (i) the filter with the mixture was placed on an LB agar plate, incubated for 3 to 6 days at 30°C; (ii) the filter was placed on an LB agar plate, incubated for 3 h at 30°C, and then the filter was transferred to an agar plate (without any nutrients) for 2 to 3 days at 30°C; (iii) the filter was placed on an agar plate for 2 to 3 days at 30°C. The mixture on the filter was resuspended in PBS and then subjected to flow cytometry using a cell sorter MoFlo XDP IntelliSort II instrument (Beckman Coulter, Denver, MA) equipped with a CyClone robotic arm for plate sorting and a 488-nm argon laser and a 70-μm nozzle orifice. Sorting of each transconjugant cell line was performed as previously described (25). Bacteria extracted from each environmental sample without donor cells were used as the negative controls. Based on the flow cytometry charts of the negative control, the gate for collecting 384 fluorescent cells was determined. Each of the 384 cells was sorted by flow cytometry, plated on LB plates, and incubated at 30°C for 2 days to allow the cells to form colonies. The 16S rRNA gene of each transconjugant (300 strains) was amplified by PCR and sequenced after the extraction of total DNA.

### PCR-Southern blot analyses.

Distribution of plasmids in different environmental samples was assessed by PCR-Southern blot analyses using a previously described method (38), with a few modifications. This analysis was performed in two steps. First, PCR with the specific primer sets for each plasmid (listed in Table S3) was performed under the following conditions: 30 cycles of 98°C for 10 s, 67°C for 5 s, and 68°C for 1 s (for trfA-F/-R and trfA_δ-F/-R); 30 cycles of 98°C for 10 s, 65°C for 5 s, and 68°C for 1 s (for repA[PromA]_F/-R); and 30 cycles of 98°C for 10 s, 57°C for 5 s, and 68°C for 1 s (for the other primer sets). The amplified DNA was then transferred to a nylon membrane filter (Hybond(r)-N+hybridization membrane; Merck KGaA, Darmstadt, Germany) using a Bio-Dot apparatus (Bio-Rad Laboratories Inc.). Probes were prepared and hybridization signals were detected using a DIG High Prime Lab/Detection Kit I (Merck KGaA, Darmstadt, Germany). The sensitivity and stringency of each probe were confirmed using positive-control DNA at different concentrations and negative-control DNA (chromosomal DNA of the recipient strains). If signals were detected, then as the second step, the amplified samples were subjected to agarose gel electrophoresis. The DNA in the gels was transferred to a nylon membrane and hybridization signals were detected again.

### Data availability.

The accession numbers deposited in DDBJ of the plasmid sequences (70 plasmids) were LC623882 to LC623932 and LC663721 to LC663740. The 16S rRNA genes of the transconjugants (transconjugants of PromA plasmids) were LC655384 to LC655683.
