# Supplementary material for: Hitherto-Unnoticed Self-Transmissible Plasmids Widely Distributed among Different Environments in Japan
Source: Appl Environ Microbiol. 2022 Sep 7;88(18):e01114-22. doi: 10.1128/aem.01114-22 (PMC9499019; doi:10.1128/aem.01114-22)
Supplement: Supplemental file 1 — Text S1 and Fig. S1 to S8. Download aem.01114-22-s0001.pdf, PDF file, 2.1 MB [file aem.01114-22-s0001.pdf]

Supplemental materials

Hitherto-unnoticed self-transmissible plasmids widely distributed among different environments in Japan

Hayakawa, Tokuda, Kaneko, Nakamichi, Yamamoto, Kamijo, Umeki et al.

Correspondence: Masaki Shintani (shintani.masaki@shizuoka.ac.jp), Ryota Moriuchi (moriuchi.ryota@shizuoka.ac.jp)

The Supplementary Materials include:

Supplemental Text S1

Figures S1-S8

Tables S1-S8 (provided in Excel format)

Supplemental Text S1

Preparation of* Escherichia coli *MG1655RGFP

pJBA28-harboring *E. coli* and helper *E. coli* with pRK2013 were grown overnight in LB with the appropriate antibiotics at 37°C and 200 rpm, and a spontaneous Rif-resistant strain of MG1655 was grown in LB with Rif at 37°C and 200 rpm. After harvesting and washing with LB, the resultant cultures were mixed and subjected to filter mating (37°C overnight). Then, the mixture on the filter was resuspended in LB and spread on an LB + Rif +Km agar plate. After incubation of the selective plates for two days, colonies with GFP fluorescence were picked and isolated. The resulting strain was named *E. coli* MG1655RGFP. The GFP gene was inserted into the *borD* gene of MG1655 (578584 nt, GenBank accession no. NC_000913) (Table 6).

Preparation of* Pseudomonas putida *KT2440G

pBSL202-harboring *E. coli* S17-1 was grown overnight in LB with the appropriate antibiotics at 37°C and 200 rpm, and *Pseudomonas putida* KT2440 was grown in LB with Tc at 30°C and 200 rpm. After harvesting and washing with LB, the resultant cultures were mixed and subjected to filter mating (30°C overnight). The mixture on the filter was resuspended in LB and spread on LB + Gm +Tc agar plates. After incubation of the selective plates for two days, colonies with Gm resistance were picked and isolated. The resultant strain was named *P. putida* KT2440G. The Gm resistance gene was inserted into PP_4780 of KT2440 (GenBank accession no. NC_002947) (Table 6).

Plasmid sequencing using next generation sequencing.

Plasmids pSM0227-02, pSM0227-07, pYK0413-01, pYK0414-12, pYK0422-04, pSN0517-01, pMH0621-02Tc, pMH0621-12, pMH0613-68, pMH0621-74, pYK0709-71, and pYK0709-104 were extracted from their hosts and sequenced using the MiSeq platform (Illumina, San Diego, CA), as described previously (1). Briefly, the plasmid DNA was fragmented using the Covaris Acoustic Solubilizer (Covaris, Woburn, MA, USA), and paired-end libraries were prepared using the TruSeq DNA PCR-Free Library Prep Kit or the TruSeq Nano DNA Library Prep Kit (Illumina). Raw sequence reads (301-bp paired-end) were filtered using Trimmomatic v. 0.39 (2) by removing adapter sequences, the last 301st base, low-quality ends (quality score, <15), and reads less than 150 bp. High-quality reads were assembled using SPAdes software (3) with a default set of k-mer sizes, and the resultant contigs were manually closed by removing the 127-bp overlapping ends. The finished sequences were confirmed by mapping the high-quality reads using BWA-MEM v. 0.7.15 (4) and visualized using the Integrative Genomics Viewer (5).

For the other plasmids, the whole genomic DNA extracted from their hosts was sequenced using the HiSeq2500 platform (Illumina). Trimmed high-quality short

reads (read length > 140 bp and quality score > 15) were assembled using SPAdes software with the plasmid option. When a circular contig(s) could not be found, the host chromosomal DNA was removed by mapping the resultant contigs on the host genome sequences [their deposited sequences, i.e., DDBJ/GenBank accession numbers NC_000913 (*E. coli* MG1655) and NC_021499 (*P. resinovorans* CA10dm4)] using Geneious software (6). The plasmids (s) were then extracted from the remaining contigs using SAMtools v. 1.7 (7) and SeqKit v.0.8.0 (8), and reassembled using SPAdes. For pMNBM077 and pMNCG080-1, gaps in plasmids were closed using *in silico* analyses, PCR, and Sanger sequencing of the PCR products.

For pMNBM065-2, pMNCF070, pMNBM072, pMNBL073, pMNCF075, pMNCF091, pMNCF092, pMNCF093-1, pMNCF093-2, pYKAM101, pYKCG107, and SMRTbell™, template library was prepared according to the instructions provided by Pacific Biosciences (Menlo Park, CA, USA). SMRT sequencing was performed using the PacBio RS II System (Pacific Biosciences). PacBio subreads were filtered (read quality > 0.8) using Bamtools v. 2.5.1 (9), and assembly of the multiplexed plasmid pools was performed using Canu v. 2.1.1 (10) with the miniReadLength=5000 setting. The resulting contigs were polished using Arrow v. 2.2.2 (https://github.com/PacificBiosciences/GenomicConsensus) and then circularized by removing artificial redundancies at the ends of the contigs. Except for pYKAM101 and pYKCG107, the high-quality short reads derived from HiSeq were aligned against the polished circular contigs using BWA-MEM, and assembly errors were corrected using Pilon v. 1.23 (11).

Preparation of mini-plasmids of IncP/P-1 plasmids and PromA plasmids.

The mini-plasmids of IncP/P-1 and PromA plasmids were constructed using the NEBuilder HiFi DNA Assembly Master Mix (New England Biotech). The DNA regions containing *ssb-trfA* and *oriV* of IncP/P-1 plasmids (several of which did not possess *ssb*) or *repA* and *oriV* or PromA plasmids were amplified by PCR with the primer sets listed in Table S3, using each plasmid as template DNA. Km or Tc resistance genes were amplified using pBBR1MCS-2 or pBBR1MCS-3 as template DNA, respectively.

Transferability of plasmids.

Filter mating assays of IncP/P-1 plasmids between *E. coli* and *Pseudomonas* strains were performed as described previously (1). The transferability of each plasmid was assessed using a mobilizable plasmid with antibiotic resistance gene (pBBR1MCS-2 or pBBR1MCS-3) as a marker. As for the recipient *E. coli*, kanamycin- or tetracycline- resistance gene was introduced into the chromosome of *E. coli* MG1655R by using pBSL202 or a mini-Tn*10* delivery vector, pBSL182 or pBSL199 (12) with strain S17-1 as described in "*Preparation of* Pseudomonas putida *KT2440G*" section. The resultant strains were named as MG1655RG, MG1655RK, MG1655RT, respectively.

Preparation of* gfp*-tagged plasmids.

For pSN1104-11*gfp*Tc, pK18mobsacB-based plasmids for homologous recombination (pK18_1104-11) were constructed using the NEBuilder HiFi DNA Assembly Master Mix (New England Biotech). Competent cells of *E. coli* JM109 (RBCBioscience) were used for transformation. A DNA region containing the $P_{A1/O4/O3}$-*gfpmut3** and *tetA* genes were amplified with primers designed using NEBuilder v1.12.17 (http://nebuilder.neb.com/) (Table S3). pJBA28 and pBBR1MCS-3 were used as template DNA. The insertion site was located in the inner region of the orf45. The 1-kb DNA regions upstream and downstream of the target sites were amplified by PCR using the primers listed in Table S3. The resultant products were inserted into the HindIII site of pK18mobsacB using NEBuilder HiFi DNA Assembly Master Mix. Competent cells of *E. coli* JM109 (RBCBioscience) were used for transformation. After homologous recombination, the nucleotide sequences on the target plasmids were determined to confirm the accurate insertion of *tetA*-$P_{A1/O4/O3}$-RBSII-*gfpmut3** into the target sites. For pSN0729-62::*gfp*, mini-Tn*5* with the Km-resistance gene and P*A1/O4/O3*-RBSII-*gfpmut3**-T_0-Cm^r-T_1 region in pJBA28 were introduced using *E. coli* S17-1*λpir*, as described previously (13, 14). The insertion site of the *gfp* cassette was determined as previously described (15).

Resistance testing.

Resistance testing for the host of each obtained IncP/P-1 plasmid (Table 3) was performed. For this testing, ampicillin (Ap, 50 μg/mL), erythromycin (Em, 25 μg/mL), chloramphenicol (Cm, 30 μg/mL), kanamycin (Km, 12.5, 25, 50 μg/mL), gentamicin (Gm, 30 μg/mL), streptomycin (Sm, 25, 50 μg/mL), and tetracycline (Tc, 12.5 μg/mL for *E. coli* and 50 μg/mL for *E. coli*) were added to Luria broth. It should be noted that the *Pseudomonas* strains are naturally resistant to Ap. The hosts of IncP/P-1 plasmids with *bla* genes including pYKBF005, pYKCT011-1, pYKBP039, pYKBL037, pYKBR041, and pYKCS045 were *P. resinovorans* CA10dm4RGFP carrying another mobilizable plasmid, pBBR1MCS-2 (with Km-resistance gene). Then each IncP/P-1 plasmid was transferred from the above strain to *E. coli* MG1655 with mini-pBBR1MCS-3 (removing the *mob* region from pBBR1MCS-3) using Ap and Tc. Subsequently, the pBBR1MCS-2 in each transconjugant was lost owing to incompatibility, and resistance testing was performed for the resultant *E. coli* strains (Table 3).

References in Supplemental Materials

1. Yanagiya K, Maejima Y, Nakata H, Tokuda M, Moriuchi R, Dohra H, Inoue K, Ohkuma M, Kimbara K, Shintani M. 2018. Novel self-transmissible and broad-host-range plasmids exogenously captured from anaerobic granules or cow manure. Front Microbiol 9:2602.

2. Bolger AM, Lohse M, Usadel B. 2014. Trimmomatic: a flexible trimmer for Illumina sequence data. Bioinformatics 30:2114–2120.

3. Bankevich A, Nurk S, Antipov D, Gurevich AA, Dvorkin M, Kulikov AS, Lesin VM, Nikolenko SI, Pham S, Prjibelski AD, Pyshkin AV, Sirotkin AV, Vyahhi N, Tesler G, Alekseyev MA, Pevzner PA. 2012. SPAdes: a new genome assembly algorithm and its applications to single-cell sequencing. J Comput Biol 19:455–477.

4. Li H. 2013. Aligning sequence reads, clone sequences and assembly contigs with BWA-MEM. arXiv 1303.3997 [q-bioGN].

5. Thorvaldsdottir H, Robinson JT, Mesirov JP. 2013. Integrative Genomics Viewer (IGV): high-performance genomics data visualization and exploration. Brief Bioinform 14:178–192.

6. Kearse M, Moir R, Wilson A, Stones-Havas S, Cheung M, Sturrock S, Buxton S, Cooper A, Markowitz S, Duran C, Thierer T, Ashton B, Meintjes P, Drummond A. 2012. Geneious Basic: an integrated and extendable desktop software platform for the organization and analysis of sequence data. Bioinformatics 28:1647–1649.

7. Li H, Handsaker B, Wysoker A, Fennell T, Ruan J, Homer N, Marth G, Abecasis G, Durbin R, 1000 Genome Project Data Processing Subgroup. 2009. The Sequence Alignment/Map format and SAMtools. Bioinformatics 25:2078–2079.

8. Shen W, Le S, Li Y, Hu F. 2016. SeqKit: A cross-platform and ultrafast toolkit for FASTA/Q fole manipulation. PLoS One 11:e0163962.

9. Barnett DW, Garrison EK, Quinlan AR, Strömberg MP, Marth GT. 2011. BamTools: a C++ API and toolkit for analyzing and managing BAM files. Bioinformatics 27:1691–1692.

10. Koren S, Walenz BP, Berlin K, Miller JR, Bergman NH, Phillippy AM. 2017. Canu: scalable and accurate long-read assembly via adaptive *k*-mer weighting and repeat separation. Genome Res 27:722–736.

11. Walker BJ, Abeel T, Shea T, Priest M, Abouelliel A, Sakthikumar S, Cuomo CA, Zeng Q, Wortman J, Young SK, Earl AM. 2014. Pilon: an integrated tool for comprehensive microbial variant detection and genome assembly improvement. PLoS One 9:e112963.

12. Alexeyev MF, Shokolenko IN. 1995. Mini-Tn*10* transposon derivatives for insertion mutagenesis and gene delivery into the chromosome of gram-negative bacteria. Gene 160:59–62.

13. Shintani M, Matsui K, Inoue J, Hosoyama A, Ohji S, Yamazoe A, Nojiri H, Kimbara K, Ohkuma M. 2014. Single-cell analyses revealed transfer ranges of IncP-1, IncP-7, and IncP-9 plasmids in a soil bacterial community. Appl Environ Microbiol 80:138–145.

14. Shintani M, Ohkuma M, Kimbara K. 2019. High-resolution comparison of bacterial conjugation frequencies. J Vis Exp https://doi.org/10.3791/57812.

15. Tokuda M, Suzuki H, Yanagiya K, Yuki M, Inoue K, Ohkuma M, Kimbara K, Shintani M. 2020. Determination of plasmid pSN1216-29 host range and the similarity in oligonucleotide composition between plasmid and host chromosomes. Front Microbiol 11:1187.

16. Wegrzyn K, Zabrocka E, Bury K, Tomiczek B, Wieczor M, Czub J, Uciechowska U, Moreno-Del Alamo M, Walkow U, Grochowina I, Dutkiewicz R, Bujnicki JM, Giraldo R, Konieczny I. 2021. Defining a novel domain that provides an essential contribution to site-specific interaction of Rep protein with DNA. Nucleic Acids Res https://doi.org/10.1093/nar/gkab113.

17. Crooks GE, Hon G, Chandonia J-M, Brenner SE. 2004. WebLogo: a sequence logo generator. Genome Res 14:1188–1190.

18. Bahl MI, Burmølle M, Meisner A, Hansen LH, Sørensen SJ. 2009. All IncP-1 plasmid subgroups, including the novel epsilon subgroup, are prevalent in the influent of a Danish wastewater treatment plant. Plasmid 62:134–139.

19. Carattoli A, Bertini A, Villa L, Falbo V, Hopkins KL, Threlfall EJ. 2005. Identification of plasmids by PCR-based replicon typing. J Microbiol Methods 63:219–228.

20. Zhang M, Visser S, Pereira e Silva MC, van Elsas JD. 2015. IncP-1 and PromA group plasmids are major providers of horizontal gene transfer capacities across bacteria in the mycosphere of different soil fungi. Microb Ecol 69:169–179.

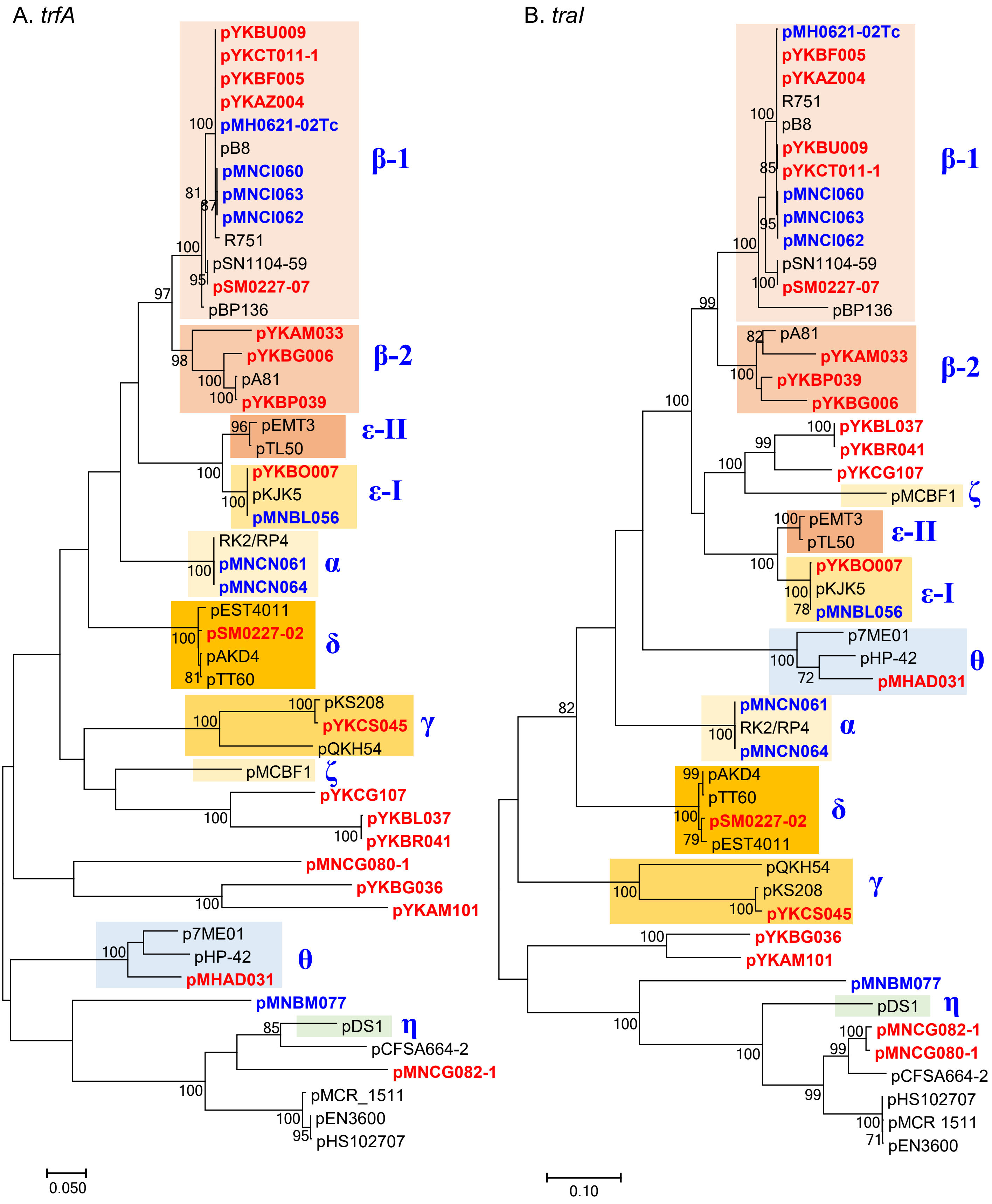

Figure S1. Phylogenetic analyses of IncP/P-1 plasmids with the reference plasmids using nucleotide sequences of *trfA* (A) and *traI* (B) and amino acid sequences of TrfA (C) and TraI (D) by maximum likelihood method with bootstrap percentages at nodes (Tamura-Nei model). A solid bar (0.050 or 0.10) shows substitutions per nucleotide position (A, B) or amino acid position (C, D). plasmids obtained by biparental mating are shown in blue, those by triparental mating are shown in red, and the other reference plasmids are shown in black.

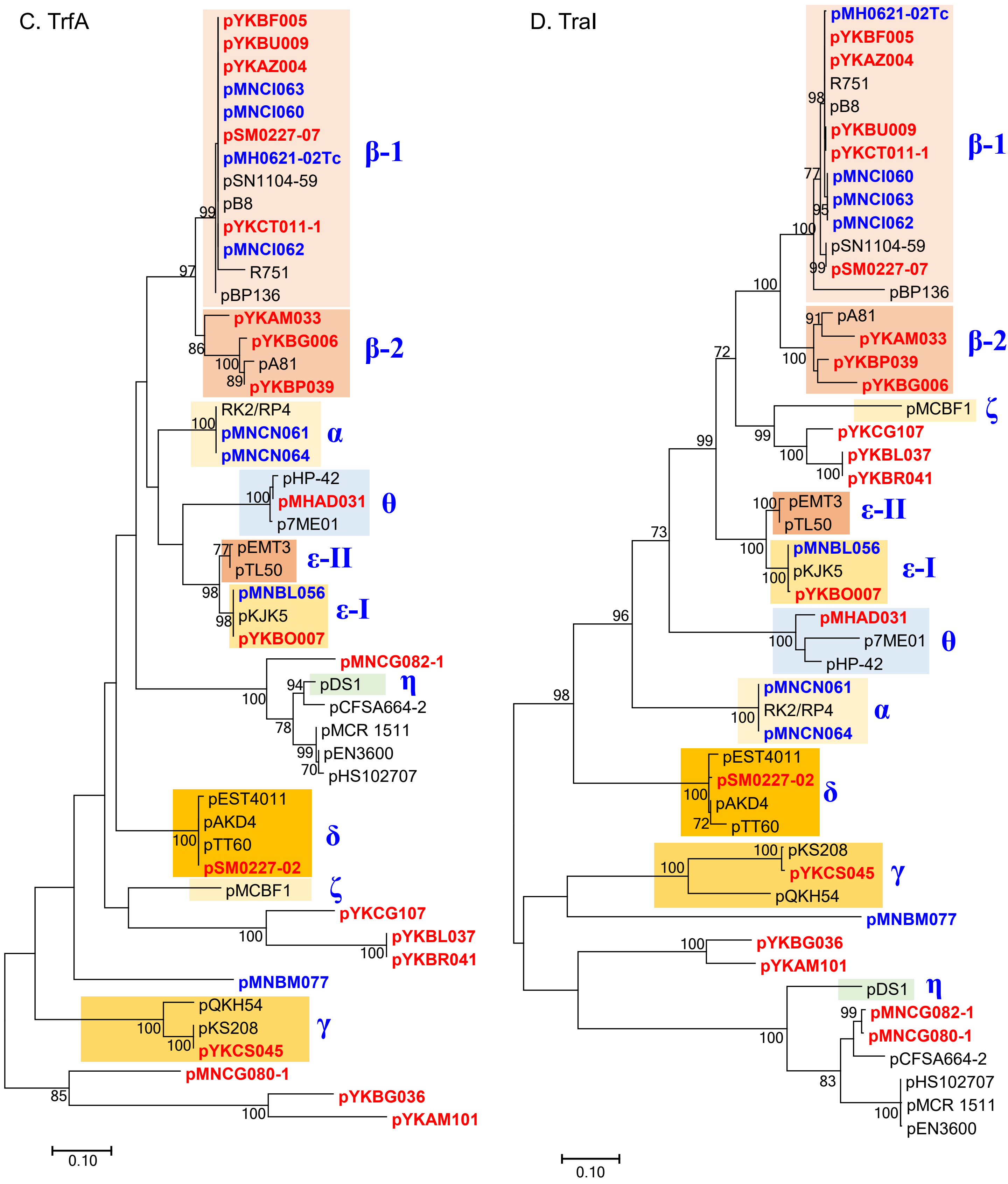

Figure S1. *continued.*

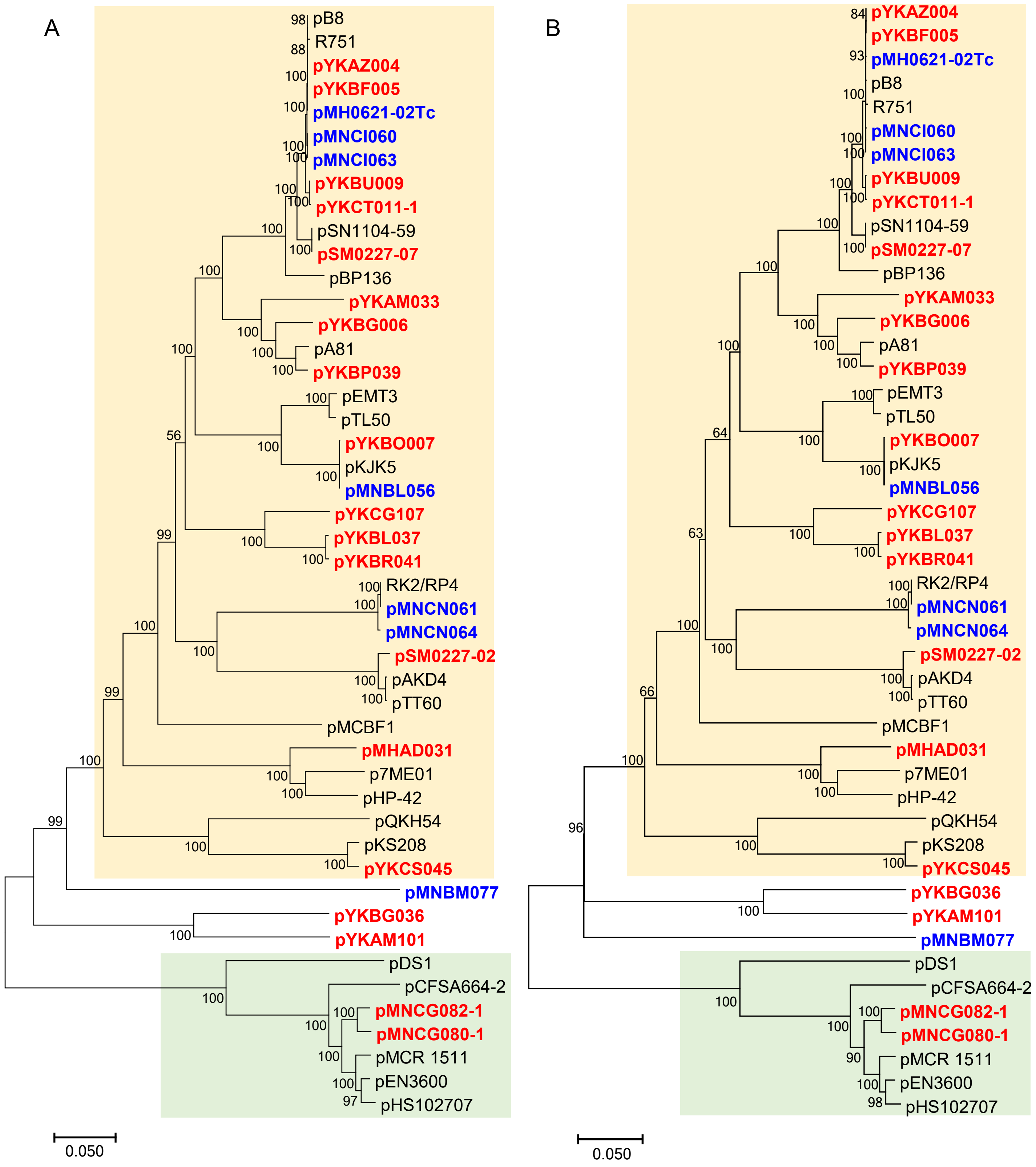

Figure S2. Phylogenetic trees of IncP/P-1 plasmids with the concatenated nucleotide sequences of 28 conserved genes (see Table 2), constructed using the neighbor-joining (a) and minimum evolutiona method (b), with bootstrap percentages at nodes; plasmids obtained by biparental mating are shown in blue, those obtained by triparental mating are shown in red, and the other reference plasmids are shown in black. The GenBank accession numbers of the reference plasmids are shown in Figure 2 legend. A solid bar (0.050) shows substitutions per nucleotide position.

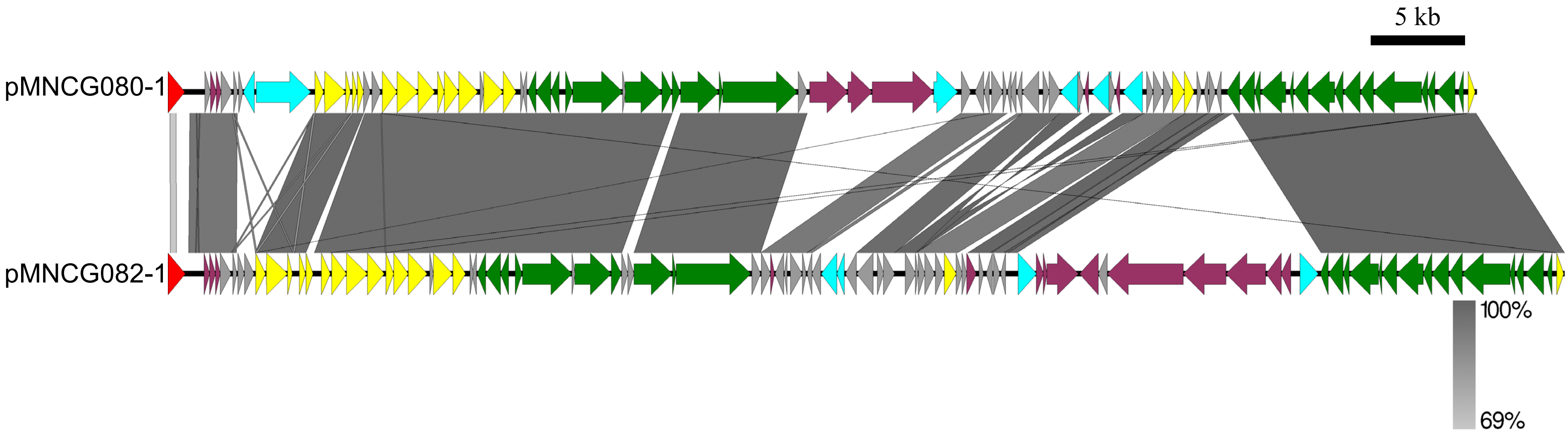

Figure S3. Alignment of pMNCG080-1 and pMNCG082-1. CDSs and their predicted functions (red for replication, green for conjugation, yellow for other genes in IncP/P-1 backbone, light blue for genes related to mobile genetic element, magenta for accessory genes, and gray for hypothetical proteins). Homologous regions are indicated by frame areas.

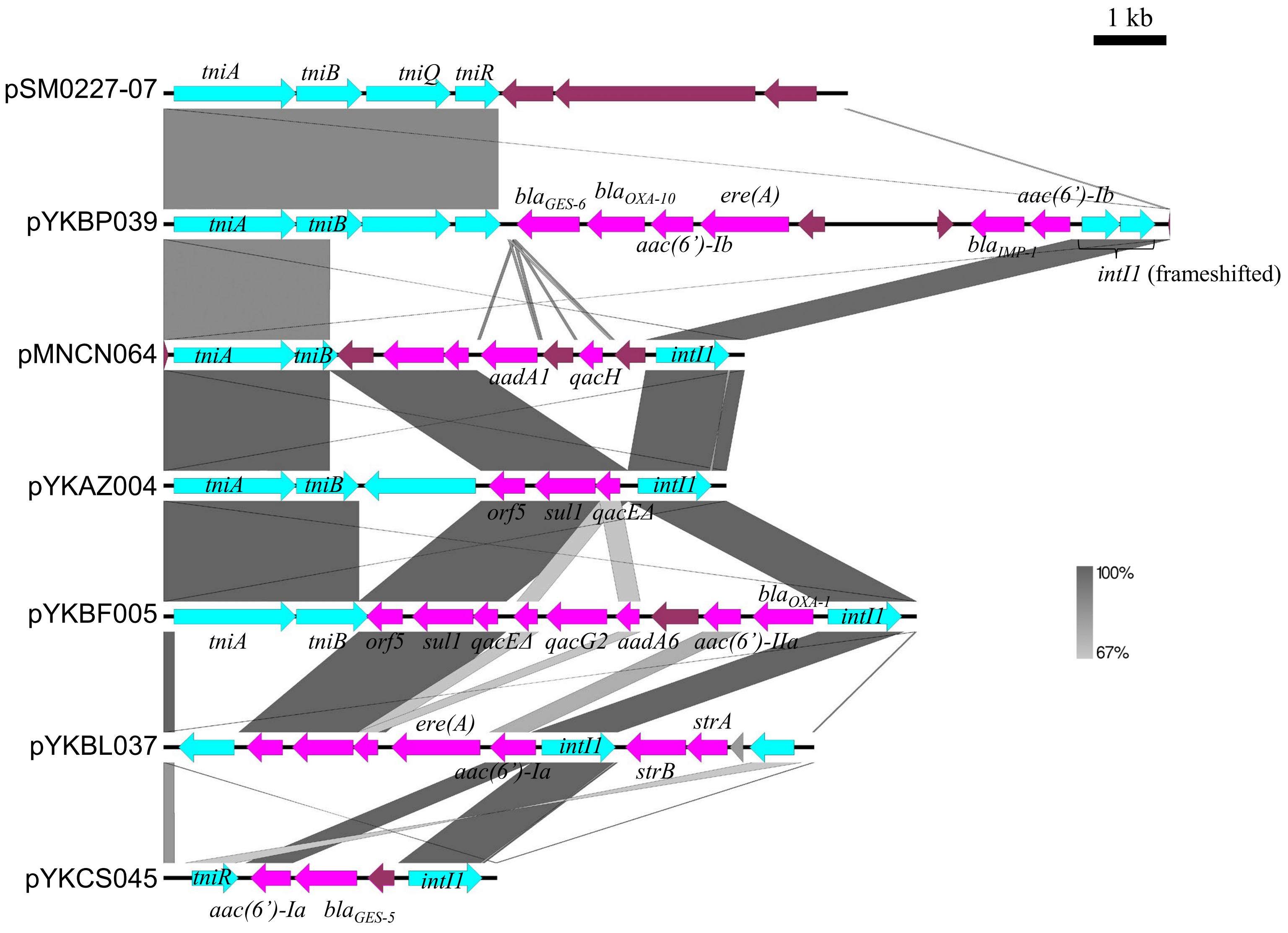

Figure S4. Comparisons of Tn*402*-like element (with *tniABOR* genes) and/or class I integron (or their remnants) found in IncP/P-1 plasmids. Coding DNA regions (CDSs) are shown, with arrows indicating their transcriptional direction. Colors indicate their putative functions: light blue: transposase or integrase related to mobile genetic elements; pink: antibiotic resistance; purple: other function. Homologous regions are indicated by frame areas.

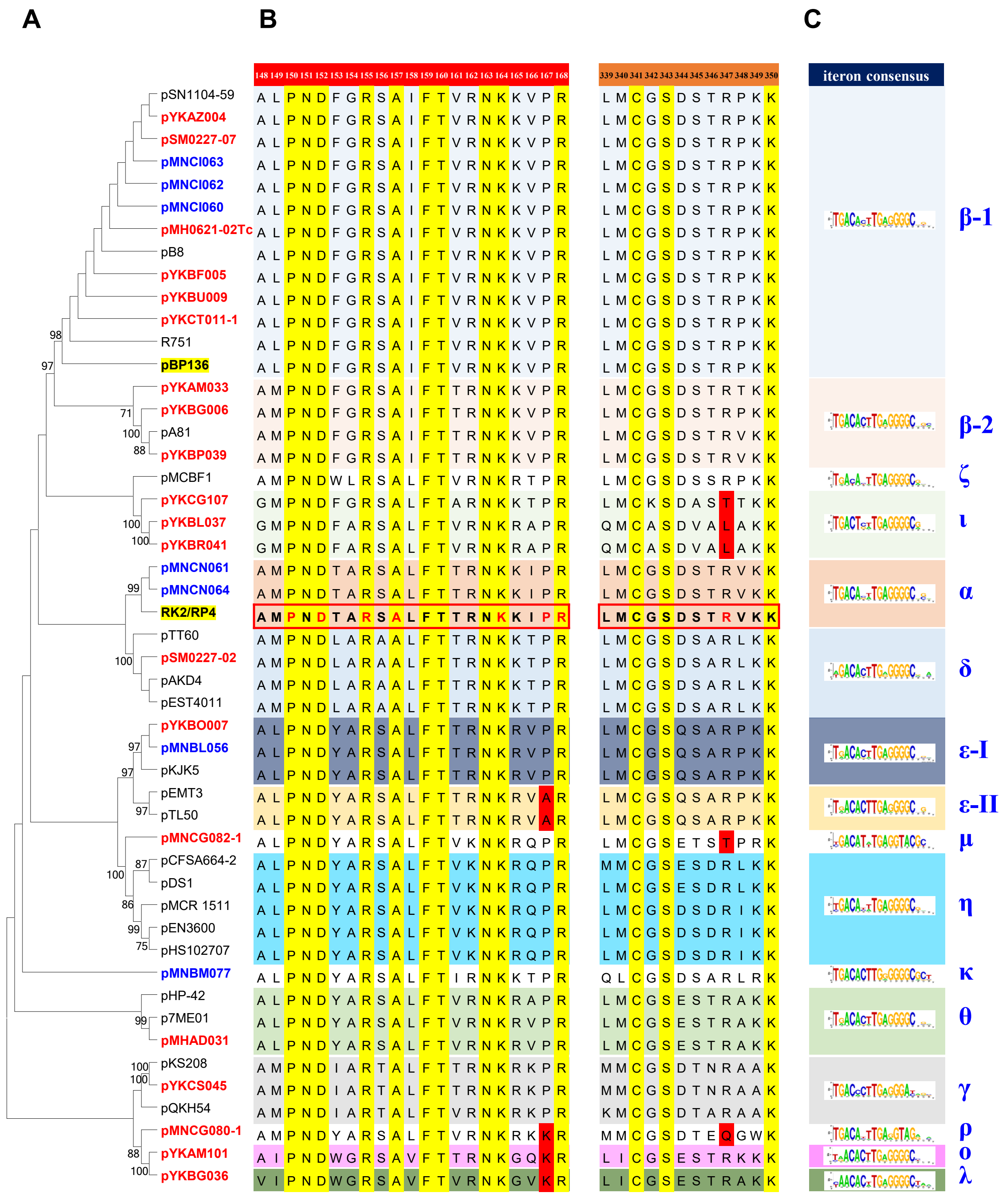

Figure S5. (A) Phylogenetic analyses of the concatenated amino acid sequences of DNA binding domain (DBD) and winged helix-turn-helix (WH) motifs of TrfA proteins of IncP/P-1 plasmids by maximum likelihood method with bootstrap percentages at nodes (Tamura-Nei model). pBP136 and RK2/RP4 are highlighted. Only the topology is shown. (B) Partial alignments of the conserved regions of DBD (148-168 aa in TrfA of RK2) and WH motifs (339-350 aa in TrfA of RK2) with the important amino acid residues interacting with iterons. The amino acid residues in TrfA of RK2 reported to affect the interaction with DNA (ref. 16) are shown in red. The conserved amino acid residues in the other IncP/P-1 plasmids are marked yellow, whereas non-conserved residues are marked red. (C) Consensus sequences of putative iterons of each IncP/P-1 subgroup made by WebLogo (ref. 17).

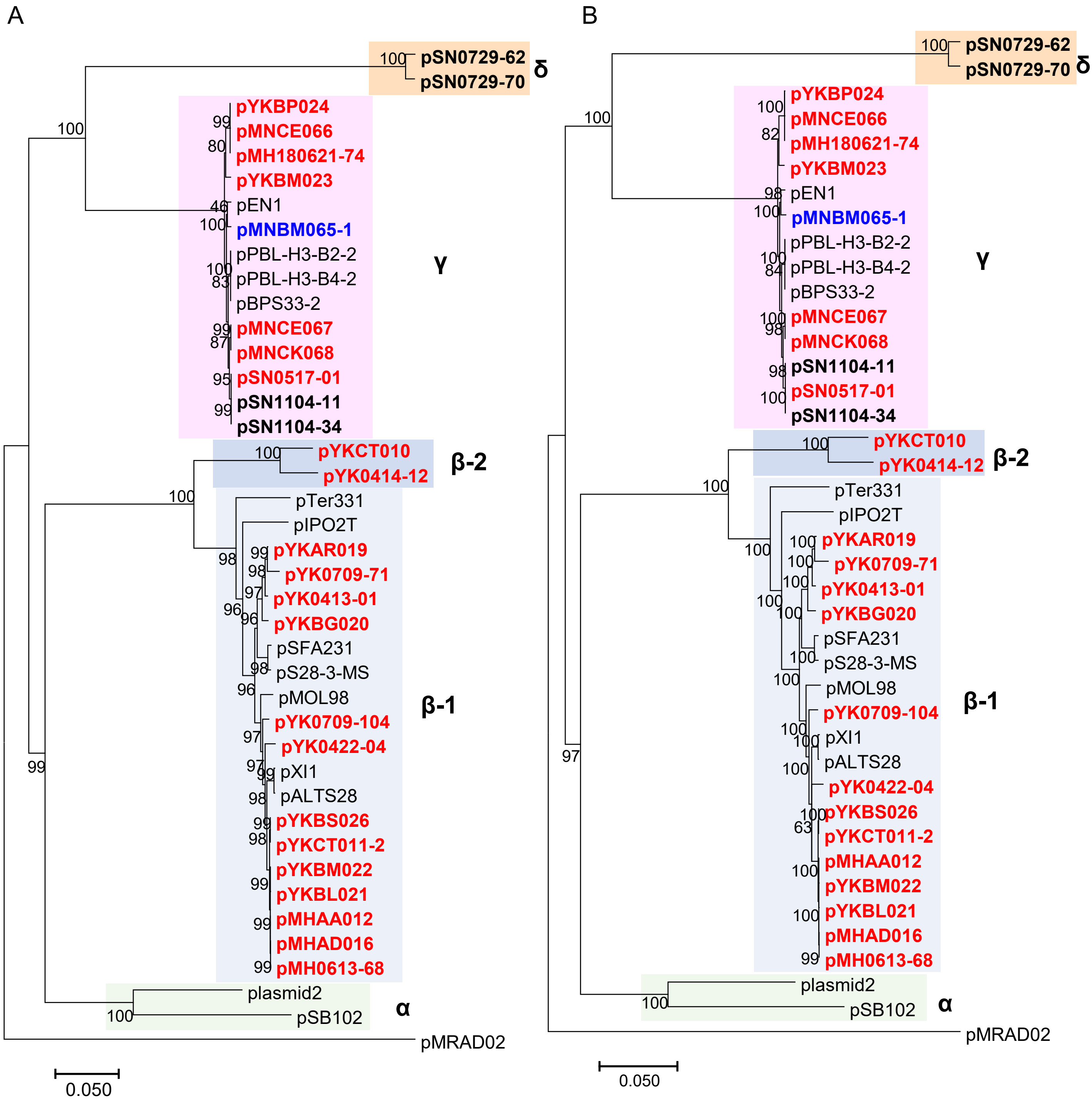

Figure S6. Phylogenetic trees of PromA plasmids with the concatenated nucleotide sequences of 24 conserved genes, constructed using the neighbor-joining (A) and minimum evolution method (B), with bootstrap percentages at nodes; a plasmid obtained by biparental mating is shown in blue, while those obtained by triparental mating are in red, and the other reference plasmids are shown in black [the plasmids obtained by our previous study (ref. 1) is shown in bold]. The GenBank accession numbers of the reference plasmids are shown in Figure 3 legend. A solid bar (0.050) shows substitutions per nucleotide position.

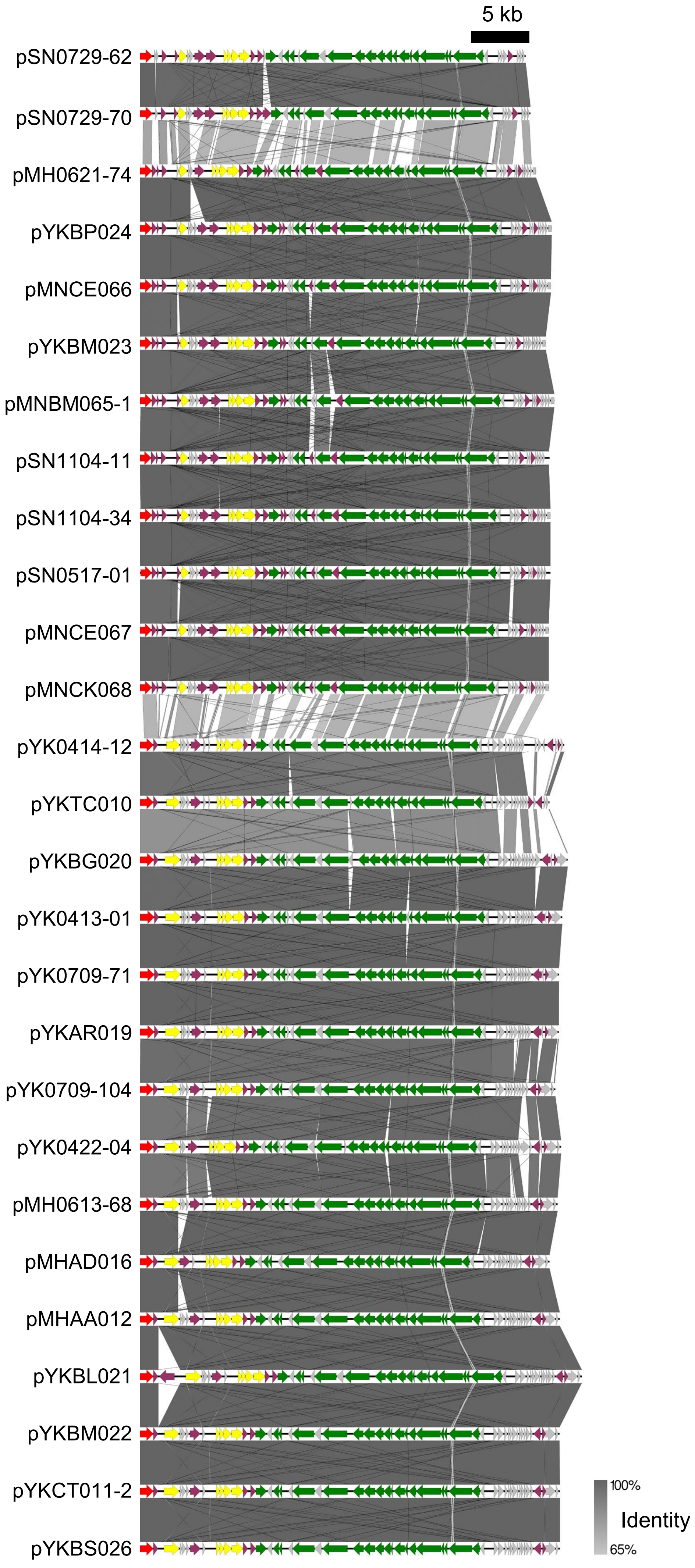

Figure S7. Comparisons of the whole genetic structure of PromA plasmids obtained in this study and our previous study (ref. 1). CDSs and their predicted functions (red for replication, green for conjugation, yellow for other conserved genes, magenta for other functional genes, gray for hypothetical proteins). Homologous regions are indicated by frame areas.

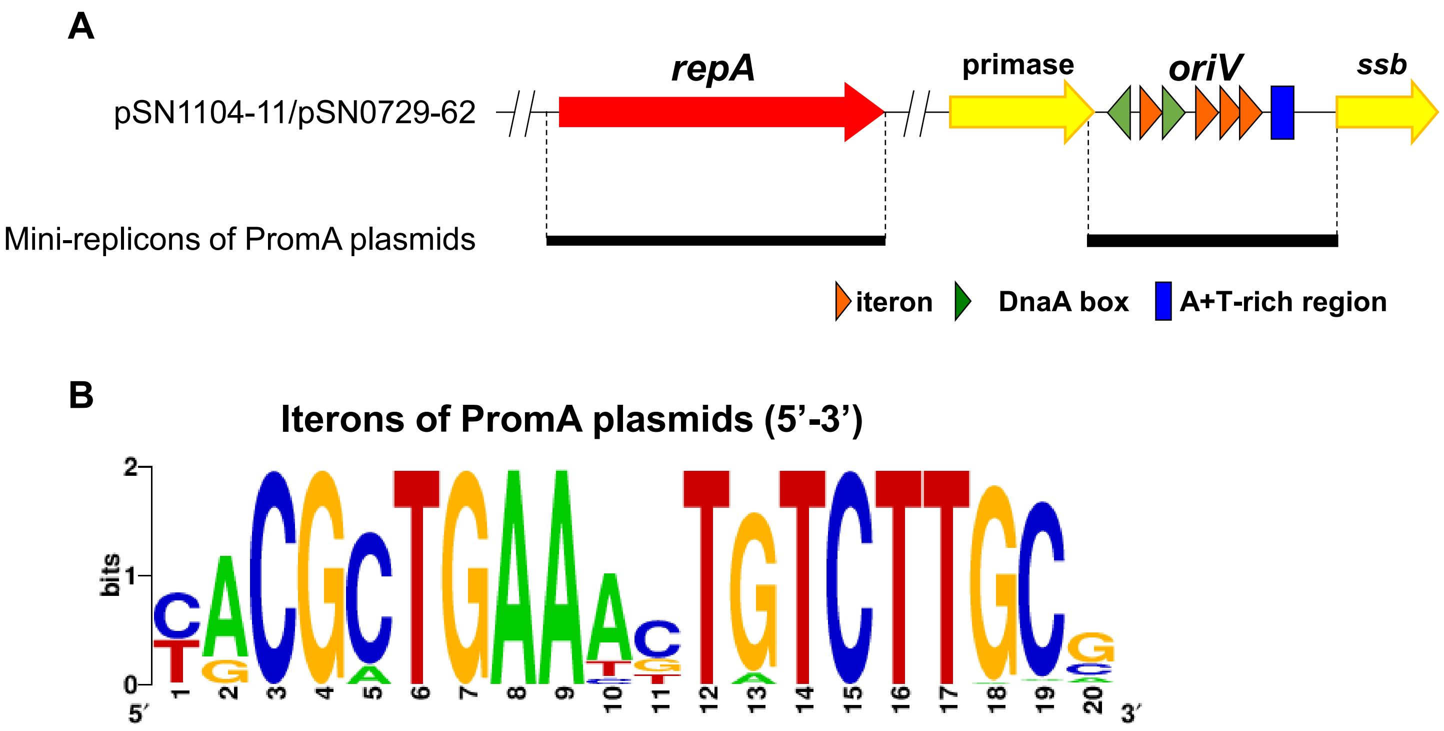

Figure S8. (A) Structure of *repA* and the *oriV* flanking region of pSN1104-11 (PromAγ) and pSN0729-62 (PromAδ). The mini-replicon of each PromA plasmid [pMH0613-68 (PromAβ-1), pYK0414-12 (PromAβ-2), pSN1104-11 (PromAγ) and pSN0729-62 (PromAδ)] was constructed by using DNA regions shown in two solid lines, *repA*, and *oriV*. Note that putative genes encoding primase and single strand DNA binding protein (Ssb) were neither conserved in pMH0613-68 (PromAβ-1) nor in pYK0414-12 (PromAβ-2). The red arrow indicates the *repA* gene, and colored triangles show putative iterons (orange) and DnaA boxes (green) with their direction. (B) Consensus sequences of putative iterons of the above four plasmids are shown [made by WebLogo (ref. 17)].
